# Zincophilic–Hydrophobic Interface Design for Dendrite-Free Aqueous Zinc-Ion Batteries

**DOI:** 10.1007/s40820-026-02153-4

**Published:** 2026-04-09

**Authors:** Yinfeng Guo, Yuxiang Xu, Yaduo Jia, Xiaoqing Zhu, Tao Zhang, Jia Zhang, Changyong Chase Cao, Qilin Gu, Guiyin Xu

**Affiliations:** 1 State Key Laboratory of Advanced Fiber Materials, College of Materials Science and Engineering, Donghua University, Shanghai, 201620 People’s Republic of China; 2 Ancan Technology LTD, Jiangsu, 214400 People’s Republic of China; 3 Tianjin Key Laboratory of Materials Laminating Fabrication and Interface Control Technology, School of Materials Science and Engineering, Hebei University of Technology, Tianjin, 300130 People’s Republic of China; 4 Department of Mechanical and Aerospace Engineering, Case Western Reserve University, Cleveland, OH 44106 USA; 5 Advanced Platform Technology (APT) Center, Louis Stokes Cleveland VA Medical Center, Cleveland, OH 44106 USA; 6 NJTECH University Suzhou Future Membrane Technology Innovation Center, Suzhou, 215100 People’s Republic of China

**Keywords:** Aqueous zinc-based batteries, Zinc anode, Interface regulation

## Abstract

Achieving Zn anode stability is critical for advancing commercialization of aqueous zinc-ion batteries. However, the instability of zinc metal anodes driven by dendritic growth, hydrogen evolution, and interfacial passivation remains a critical obstacle for advancing aqueous zinc-ion batteries. In this paper, we report a synergistic interfacial engineering strategy that integrates in situ-grown zincophilic copper nanorod arrays with a self-assembled layer of 1-dodecanethiol to regulate ion flux and suppress side reactions simultaneously. The water-poor electric double-layer microenvironment derived from this dual-function “zincophilic–hydrophobic” architecture (denoted as HS-Cu@Zn) promotes uniform Zn deposition along the (100) plane, enhances desolvation kinetics (Zn^2^^+^ transference number increased from 0.47 to 0.75), and effectively excludes electroactive water molecules from the anode surface. As a result, the symmetric cells exhibit ultra-long cycling stability over 3500 h at 1 mA cm^−2^, while Zn||Cu half-cells maintain a Coulombic efficiency of 99.65% for 900 cycles. ZnVO||HS-Cu@Zn full cell demonstrates exceptional cycling stability, achieving 2000 stable cycles at 5 A g^−1^ with an average Coulombic efficiency of 99.8%.

**Supplementary Information:**

The online version contains supplementary material available at 10.1007/s40820-026-02153-4.

## Highlights

- The loose rough surface of the Cu nanorod arrays provided ordered zinc migration channels and was used as a conductive substrate to modulate the nucleation sites of Zn^2+^, thereby promoting the uniform deposition of Zn^2+^.
- The self-assembled layer of thiol molecules effectively reduces the concentration of water molecules at the interface of the Zn anode, ensuring the stability and reversibility of the Zn anode.
- The synergistic interfacial engineering strategy reduced the desolvation energy of zinc ions and achieved a higher ion transfer number.

**Supplementary Information:**

The online version contains supplementary material available at 10.1007/s40820-026-02153-4.

## Graphical abstract

**Figure:**
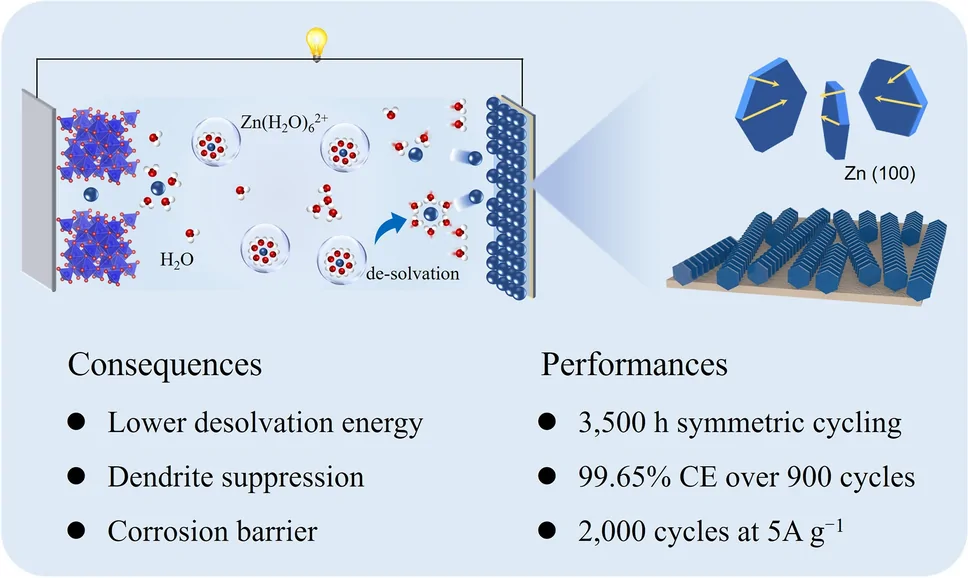

## Introduction

Aqueous zinc-ion batteries (AZIBs) have emerged as promising candidates for next-generation secondary batteries, owing to their intrinsic safety, environmental benignity, low cost, and high theoretical specific capacities (820 mAh g^−1^ and 5855 mAh cm^−3^) of zinc metal anodes [1–3]. However, in aqueous electrolytes, the Zn anode faces several challenges: corrosion and hydrogen evolution reaction (HER) caused by electrochemically active water molecules, as well as dendrite growth due to uneven Zn^2+^ deposition resulting from irregular electric field distribution and localized nucleation. These interfacial issues drastically limit the cycle life and Coulombic efficiency (CE) of zinc-ion batteries [4, 5], significantly hindering the practical development of metal–water-based Zn batteries. To address these challenges, multiple strategies have been proposed, including electrolyte engineering [6–8], functional separators design [9, 10], and interfacial modification of Zn anodes through protective coatings [11, 12] or structural tuning [13]. Among them, constructing engineered interfacial layers has emerged as a highly effective and scalable approach to stabilize Zn deposition by regulating ion flux and suppressing side reactions. Various hydrophilic coatings, such as ZnF_2_ [14], kaolinite [15], barium titanate [16], and organic polymers [17], have been shown to facilitate Zn nucleation and mitigate dendrite growth. In addition, the use of zincophilic metals or alloys (e.g., Sn, In, Sb) has demonstrated reduced Zn nucleation barriers and improved plating uniformity through better electric field homogenization [18–20].

It is worth noting that electrochemically active water molecules are also a central factor affecting the stability of Zn anodes in aqueous batteries. Typically, the metal-modified material has a nanoscale rough structure, consisting of particles or flakes, which provides an easy access for free water molecules to the zinc metal surface [21–23]. As a result, the enrichment of free water molecules at the Zn anode–electrolyte interface can lead to electrochemical corrosion of the zinc metal over a long period of time, thus accelerating the formation of the irreversible product “dead zinc” [24]. At the same time, hydrogen evolution accelerates the detachment of the surface-modified material to the point where zinc utilization and reversibility are reduced. Thus, a rationally designed interface that simultaneously promotes uniform Zn plating and suppresses interfacial water activity is urgently needed

**Fig. 1:**
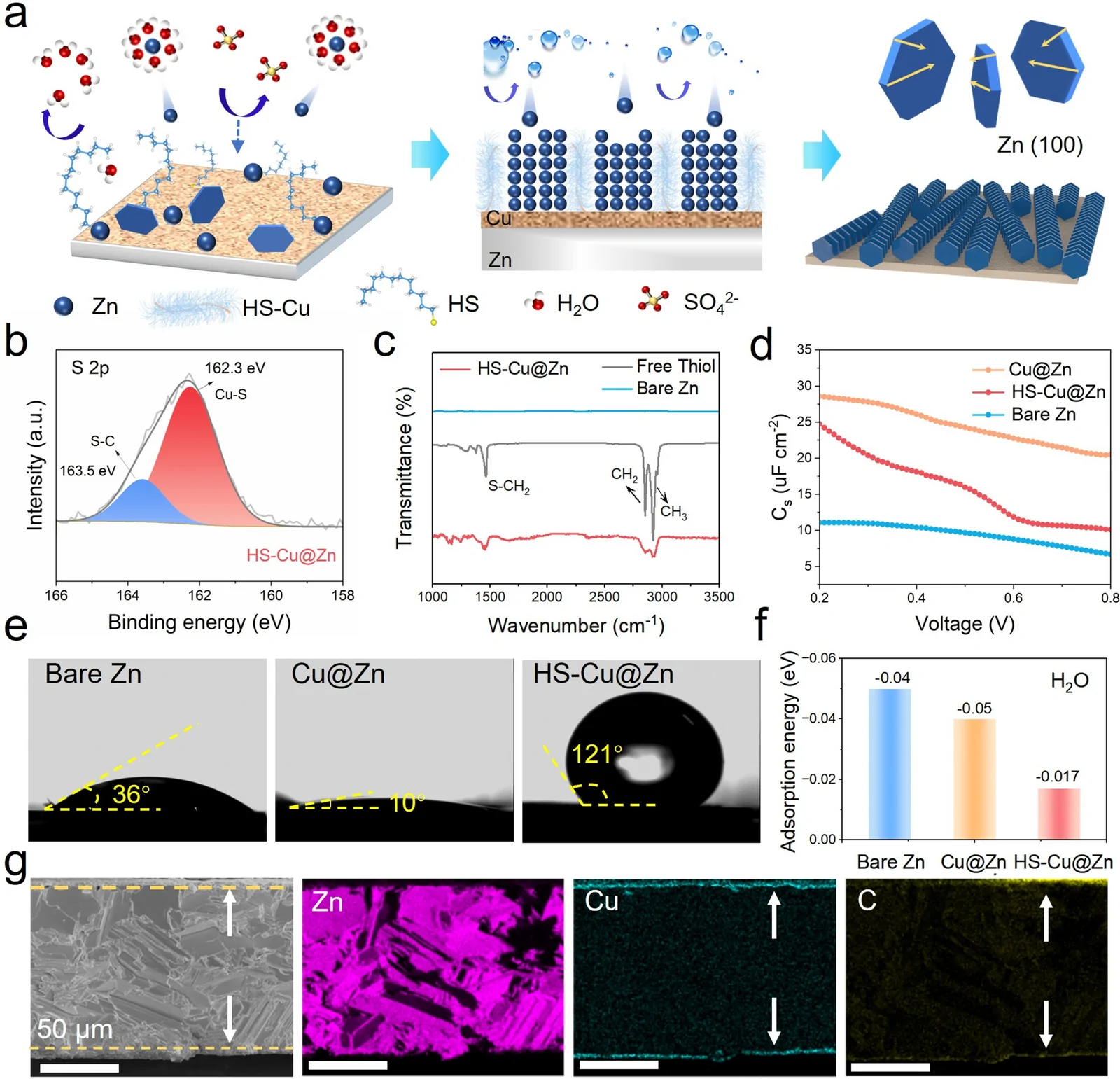
Construction and characterization of the engineered "zincophilic–hydrophobic" interface on Zn anode (HS-Cu@Zn). **a** Schematic illustration of the HS-Cu@Zn interface architecture showing in situ-grown Cu nanorod arrays and self-assembled thiol layer, enabling uniform Zn^2+^ deposition and suppression of interfacial side reactions. **b** High-resolution XPS spectra of S 2*p* for HS-Cu@Zn. **c** FTIR spectra of bare Zn, HS-Cu@Zn, and free thiol molecules, highlighting the chemical signature of the self-assembled thiol layer on the modified anode. **d** Differential capacitance curves in Na_2_SO_4_ electrolyte. **e** Water contact angle measurements on bare Zn, Cu@Zn, and HS-Cu@Zn surfaces. **f** DFT-calculated adsorption energies of H_2_O molecules on bare Zn, Cu@Zn and HS-Cu@Zn surfaces. **g** SEM image and corresponding cross-sectional EDS elemental mapping of HS-Cu@Zn

Self-assembled layers offer a promising route to achieve such multifunctional interfacial engineering. This molecular layer forms via spontaneous chemisorption on metal surfaces, enabling precise control over surface chemistry, wettability, and interfacial interactions [25–28]. Their application has been extensively explored in biosensors and nanotechnology, but their integration with metal anode systems, particularly in aqueous batteries, remains underexplored [29, 30]. Based on this, we introduce a synergistic interfacial design that combines in situ-grown copper nanorod arrays as zincophilic channels with a hydrophobic layer of 1-dodecanethiol on the Zn surface (Fig. 1a). The copper nanorods regulate nucleation and reduce charge transfer resistance, while the thiol layer, covalently bonded via Cu–S interactions, repels free water molecules. This engineered “zincophilic–hydrophobic” interface enhances desolvation of hydrated Zn^2+^ and promotes planar Zn deposition along the (100) plane, inhibits dendritic growth, and significantly improves interfacial stability. As a result, the symmetric cell with HS-Cu@Zn exhibits a stable cycle up to 3500 h at 1 mA cm^−2^. ZnVO||HS-Cu@Zn full cell was able to stably cycle for 2000 cycles at a high current density of 5 A g^−1^ with an average Coulombic efficiency of 99.8%. Our findings offer a generalizable strategy for interface engineering in aqueous metal batteries by simultaneously addressing dendrite formation and water-induced degradation through dual-function interfacial modulation.

## Experimental Section

### Synthesis of Cu@Zn and HS-Cu@Zn Anodes

Commercial zinc foil (100 μm, 4 cm × 5 cm) was cleaned via ultrasonic treatment in ethanol to remove surface contaminants. To fabricate Cu@Zn, the cleaned foils were immersed in an aqueous solution containing 0.2 M CuSO_4_ (Aladdin Biochemical Technology Co., Ltd. AR, 99%) and 0.1 M H_2_SO_4_ (Sinopharm Chemical Reagent Co., Ltd. GR) for 5s, allowing galvanic replacement. After thorough rinsing with deionized water, the samples were dried in a vacuum oven at 50 °C for 1h. For hydrophobic surface modification, the dried Cu@Zn was immersed in a 1-dodecanethiol solution for 30 min to allow the formation of a self-assembled layer. The resulting HS-Cu@Zn anodes were sequentially rinsed with ethanol and deionized water and dried under vacuum*.*

### Synthesis and Fabrication of ZnVO Cathodes

Zn_0.25_V_2_O_5_ (ZnVO) was synthesized via a hydrothermal method. Specifically, 0.5456 g of V_2_O_5_ (Aladdin Biochemical Technology Co., Ltd. ≥ 99.5%) and 0.4275 g of zinc acetate (Aladdin Biochemical Technology Co., Ltd. ≥ 99.99% metals basis) were dissolved in 67 mL of deionized water. 4.66 mL acetone (Sinopharm Chemical Reagent Co., Ltd. CP, 99.0%) and concentrated 0.3 mL HNO_3_ (Sinopharm Chemical Reagent Co., Ltd. AR) were added, and the mixture was stirred for 2h. The solution was then sealed in a 100 mL Teflon-lined stainless-steel autoclave and heated at 180 °C for 24h. The resulting dark green powder was washed with water and ethanol, followed by drying at 80 °C under vacuum overnight. To fabricate the cathode, ZnVO, acetylene black, and polyvinylidene fluoride (PVDF) were mixed in a weight ratio of 7:2:1 and dispersed in 1-methyl-2-pyrrolidinone (NMP, Shanghai Titan Technology Co., Ltd. 99.5%) to form a uniform slurry. The slurry was coated onto graphite paper and dried in a vacuum oven at 60 °C overnight. The active material loading was approximately 1.5 mg cm⁻^2^.

## Results and Discussion

### Hierarchical Interface Engineering and Structural Characterization of HS-Cu@Zn Anodes

To realize an anode surface that simultaneously directs Zn^2+^ migration and suppresses parasitic reactions, a zincophilic–hydrophobic interface was fabricated via a two-step strategy. First, copper nanorod arrays were grown in situ onto zinc foil through a rapid galvanic replacement reaction, forming the Cu@Zn substrate (Figs. S1 and S2). Subsequently, 1-dodecanethiol molecules were self-assembled onto the Cu nanorods to yield a monomolecular hydrophobic coating, creating the HS-Cu@Zn anode. This hierarchical structure is designed to synergistically regulate zinc nucleation behavior and reduce the concentration of water molecules at the anode interface, promoting uniform zinc-ion deposition to enhance the stability of the zinc anode (Fig. 1a).

The high-resolution X-ray photoelectron spectroscopy (XPS) spectrum of Cu 2*p* in Cu@Zn anode was deconvolved into six peaks. The peaks at binding energies of 932.6 and 952.4 eV correspond to the Cu 2*p*_3/2_ and Cu 2*p*_1/2_ of Cu (0), respectively [31]. The peaks at 934.9 and 954.8 eV are attributed to the Cu 2*p*_3/2_ and Cu 2*p*_1/2_ of Cu^2+^ and are accompanied by characteristic oscillating satellite peaks at 942.7 and 962.5 eV, which are indicative of Cu^2+^ [32, 33] (Fig. S3). After thiol assembly, the Cu^2+^ peak disappeared and the Cu (0) peak (931.9–951.7 eV) shifted slightly due to Cu–S bond formation. This change confirms that the binding of sulfur can significantly change the charge distribution around the Cu atoms, which can effectively enhance the electron transfer during the reaction [34]. The S 2*p* spectrum of HS-Cu@Zn (Fig. 1b) showed peaks at 162.3 and 163.5 eV, corresponding to Cu–S and C–S bonds, respectively. Compared to the Cu@Zn anode, the characteristic peaks of S as well as the peak changes of Cu elements can be clearly observed on the HS-Cu@Zn surface (Fig. S4), confirming covalent bonding of thiol groups to the Cu nanostructure. Fourier transform infrared spectroscopy (FTIR, Fig. 1c) further corroborated the molecular identity of the assembled layer. In contrast to free thiols, the C–H stretching vibration peaks of –CH_3_ and –CH_2_ groups appear on the surface of HS-Cu@Zn, indicating that the sulfhydryl group in the thiol molecule binds to the Cu metal through dehydrogenation. In fact, the sulfur atoms in the thiol molecule have lone pair of electrons that are able to form coordination bonds with the empty orbitals on the surface of metallic copper and change the chemical properties of the Cu surface [35, 36]. To verify that the self-assembled molecular layer remains firmly anchored to the Cu nanolayer after battery assembly, electrochemical double-layer capacitance measurements in Na_2_SO_4_ electrolyte (Fig. 1d) revealed that the initial interfacial capacitance of the HS-Cu@Zn surface differs from that of bare Zn and Cu@Zn surfaces, indicating that the thiol self-assembled layer alters the interfacial charge distribution [37]. The interaction between the main chains of the thiol molecule ensures an efficient stacking of the thiol layer and the outward arrangement of the long-chain alkyl groups gives it hydrophobicity.

The hydrophobicity of HS-Cu@Zn can be visualized by the contact angle test of the H_2_O on different Zn anode surfaces. The contact angle of bare zinc (36°) is much smaller than that of HS-Cu@Zn (121°). It is worth noting that due to the formation of Cu nanorod arrays increases the roughness of the zinc surface, while the contact angle of Cu@Zn surface is 10° (Fig. 1e). Meanwhile, the adsorption energy of water molecules on the surface of Zn anode was calculated to reveal the protection mechanism of self-assembled thiol molecular layer on Zn anode. As depicted in Fig. 1f, the adsorption energy of water molecules on the HS-Cu@Zn surface is − 0.017 eV, which is significantly lower than that on the bare Zn (− 0.05 eV) and Cu@Zn (− 0.04 eV) surfaces. Therefore, the dense modification of the thiol self-assembled monolayer anchored by the Cu nanolayer can modulate the hydrophilicity of the rough Cu@Zn surface, thereby mitigating the corrosive effect of water molecules on the zinc anode interface.

Scanning electron microscopy (SEM) showed that Cu@Zn and HS-Cu@Zn had dramatically increased surface roughness compared to bare Zn (Fig. S5), due to the formation of densely packed “wheat-like” Cu nanorods. After thiol assembly, additional nanoflower-like structures were observed, likely corresponding to the organized thiol layer (Fig. S6). Energy-dispersive X-ray spectroscopy (EDS) revealed a uniform distribution of Zn, Cu, S, and C elements across the HS-Cu@Zn surface (Fig. S7). In addition, cross-sectional EDS (Fig. 1g) revealed a ~ 4 µm thick surface layer composed of Cu and C elements on top of the Zn substrate, highlighting the successful construction of the bilayer interface. These results validate the design of a chemically anchored, zincophilic and hydrophobic nanostructured surface, which is expected to suppress parasitic side reactions while guiding uniform Zn^2+^ deposition.

### ***Desolvation Kinetics and Zn***^***2+***^*** Deposition Behavior on the HS-Cu@Zn Interface***

**Fig. 2:**
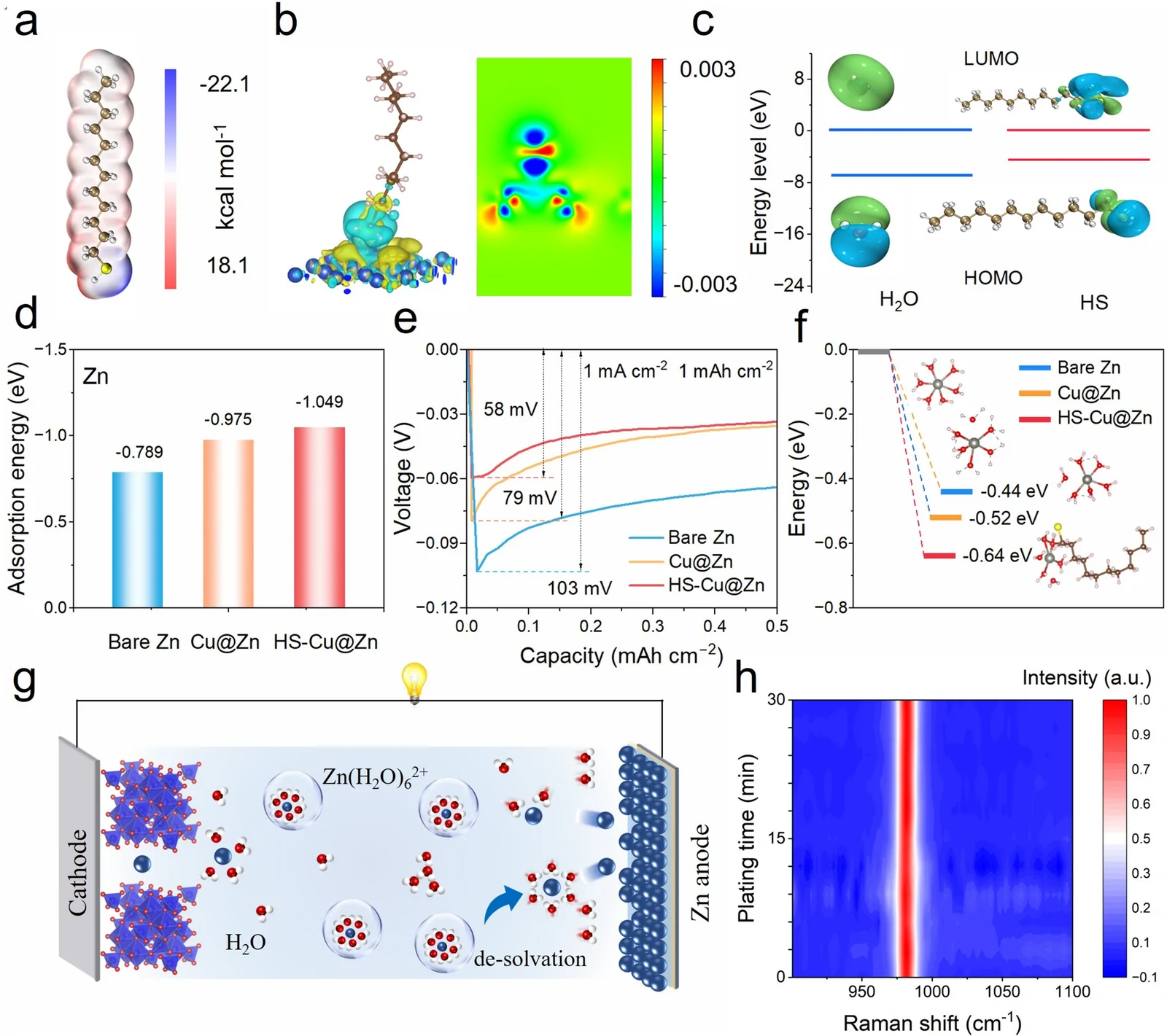
Theoretical and experimental analysis of interfacial desolvation kinetics and Zn^2+^ deposition behavior on engineered HS-Cu@Zn anode. **a** ESP distribution of 1-dodecanethiol (HS). **b** Charge density difference map illustrating strong chemisorption between Cu nanolayers and HS molecules, with electron transfer from sulfur to copper. **c** HOMO and LUMO energy levels of HS and H_2_O molecules. **d** DFT-calculated Zn atom adsorption energies on bare Zn, Cu@Zn, and HS-Cu@Zn surfaces. **e** Nucleation overpotentials during Zn deposition on bare Zn, Cu@Zn, and HS-Cu@Zn substrates at 1 mA cm⁻^2^ in 2 M ZnSO_4_. **f** DFT-calculated desolvation energies for hydrated Zn(H_2_O)_6_^2+^ complexes on different interfaces. Inset: corresponding molecular models. **g** Schematic illustration of Zn^2+^ desolvation and diffusion at the HS-Cu@Zn electrode/electrolyte interface. **h** In situ Raman spectra tracking SO_4_^2^⁻ signal evolution on HS-Cu@Zn anode during Zn deposition

To elucidate how the engineered interface regulates Zn^2+^ transport and deposition, we conducted a combined theoretical and electrochemical analysis. Density functional theory (DFT) calculations, electrostatic potential (ESP), and molecular orbital analysis were performed to understand the chemical interactions between Zn^2+^ ions, water molecules, and the surface. Figures S8 and 2a show the water molecule and a 1-dodecanethiol molecule, with the oxygen atom being the main active site of negative charge in the water molecule. In contrast, the active site of the sulfhydryl molecule is concentrated on the sulfur atom with a high negative electrostatic potential, which indicates that –SH is highly reactive and covalently binds to Cu [38]. The differential charge density distribution further verifies the strong chemisorption of thiol molecules on Cu nanolayers, demonstrating preferential electron transfer from HS groups to Cu during this interfacial process (Fig. 2b). The reconstructed zinc anode surface effectively modulates the electric double-layer (EDL) charge distribution while suppressing interfacial water adsorption, consequently reducing the EDL capacitance from 98.5 to 25.6 μF cm^−2^ (Fig S9) [39]. Additionally, HS shows high HOMO energy level (− 6.35 eV) and low LUMO energy level (0.32 eV) compared to water molecules (− 8.52 and 0.42 eV), which proves that –SH groups in thiol are more susceptible to lose electrons and tightly adhere to the Cu nanolayer surface, which effectively inhibits the decomposition of water molecules and stabilizes the Zn anode interface (Fig. 2c).

DFT simulations were used to calculate the adsorption energy of a Zn atom on various surfaces to explain the intrinsic driving force of the accelerated desolvation of Cu nanolayers. At the electrode/electrolyte interface, the stronger zincophilicity facilitates the zinc ions to easily overcome the solvated structural barriers and accelerate the reaction process. HS-Cu@Zn exhibited the strongest Zn binding (− 1.049 eV), significantly larger than bare Zn surface (− 0.789 eV). Notably, the adsorption energy of a Zn atom on the Cu@Zn surface (− 0.975 eV) was intermediate between that on the bare Zn and HS-Cu@Zn (Figs. 2d and S10), indicating that the zincophilic Cu nanorods and self-assembled thiol layer work cooperatively to lower the nucleation energy barrier and promote Zn^2+^ capture. Meanwhile, nucleation overpotentials were evaluated by galvanostatic plating tests. As shown in Fig. 2e, the initial nucleation overpotential for HS-Cu@Zn was only 58 mV, significantly lower than that the Cu@Zn (79 mV) and the bare Zn (103 mV), confirming that the modified interface facilitates easier nucleation of Zn. It is obvious that the good zincophilicity of the in situ-grown Cu nanolayers and the excellent interfacial desolvation ability of the self-assembled thiol layer led to the uniform deposition of Zn ions. DFT simulations also evaluated the desolvation energetics of hydrated Zn^2+^. The removal energy for a single water molecule from Zn(H₂O)_6_^2+^ was calculated to be -0.64 eV on HS-Cu@Zn, which is lower than the energies on Cu@Zn (− 0.52 eV) and bare Zn (− 0.44 eV) (Figs. 2f and S11). Therefore, Cu nanolayers with abundant zincophilic sites provided ultrafast ion diffusion channels, and the self-assembled thiol hydrophobic layer modulated the desolvation kinetics at the Zn anode interface and suppressed the corrosion and hydrogen evolution caused by the decomposition of electroactive water molecules to increase the interfacial stability (Fig. 2g). Theoretical simulations and experimental results verify that the combination of the zincophilic ability of Cu nanolayers and the thiol hydrophobic layer facilitates the removal process of zinc ions from [Zn(H_2_O)_6_]^2+^ at the anode/electrolyte interface and reduces the energy barrier for desolvation. Meanwhile, the abundant nanoscale diffusion pathways enable the rapid and continuous transfer of Zn^2+^ and ensure uniform Zn^2+^ flux.

The dynamic evolutionary behavior of the EDL at the zincophilic–hydrophobic interface of Zn^2+^ was monitored in real time by in situ Raman spectra to investigate the mechanism of its influence on the Zn deposition process. In fact, in the aqueous ZnSO_4_ electrolyte, the coordination number of SO_4_^2−^ in the coordination structure of Zn^2+^ is about 0.8 ~ 1, and the Raman signal intensity is positively correlated with the ion concentration [40]. Therefore, by tracing the characteristic peak and the intensity change of SO_4_^2−^ at 980 cm^−1^, the concentration distribution state of Zn^2+^ at the interface can be indirectly characterized [41]. For the bare Zn anode, the intensity of the SO_4_^2−^ Raman signals continuously change during the dynamic process of Zn deposition (Fig. S12), indicating that the Zn^2+^ flux exhibits uneven characteristics at the interface. In contrast, the HS-Cu@Zn anode maintained a stable Raman response throughout the deposition process (Fig. 2h), confirming that its interfacial Zn^2+^ concentration was uniformly distributed and the dynamic equilibrium was controllable, resulting in a homogeneous Zn deposition.

### Electrochemical Kinetics and Corrosion Resistance of HS-Cu@Zn Anodes

**Fig. 3:**
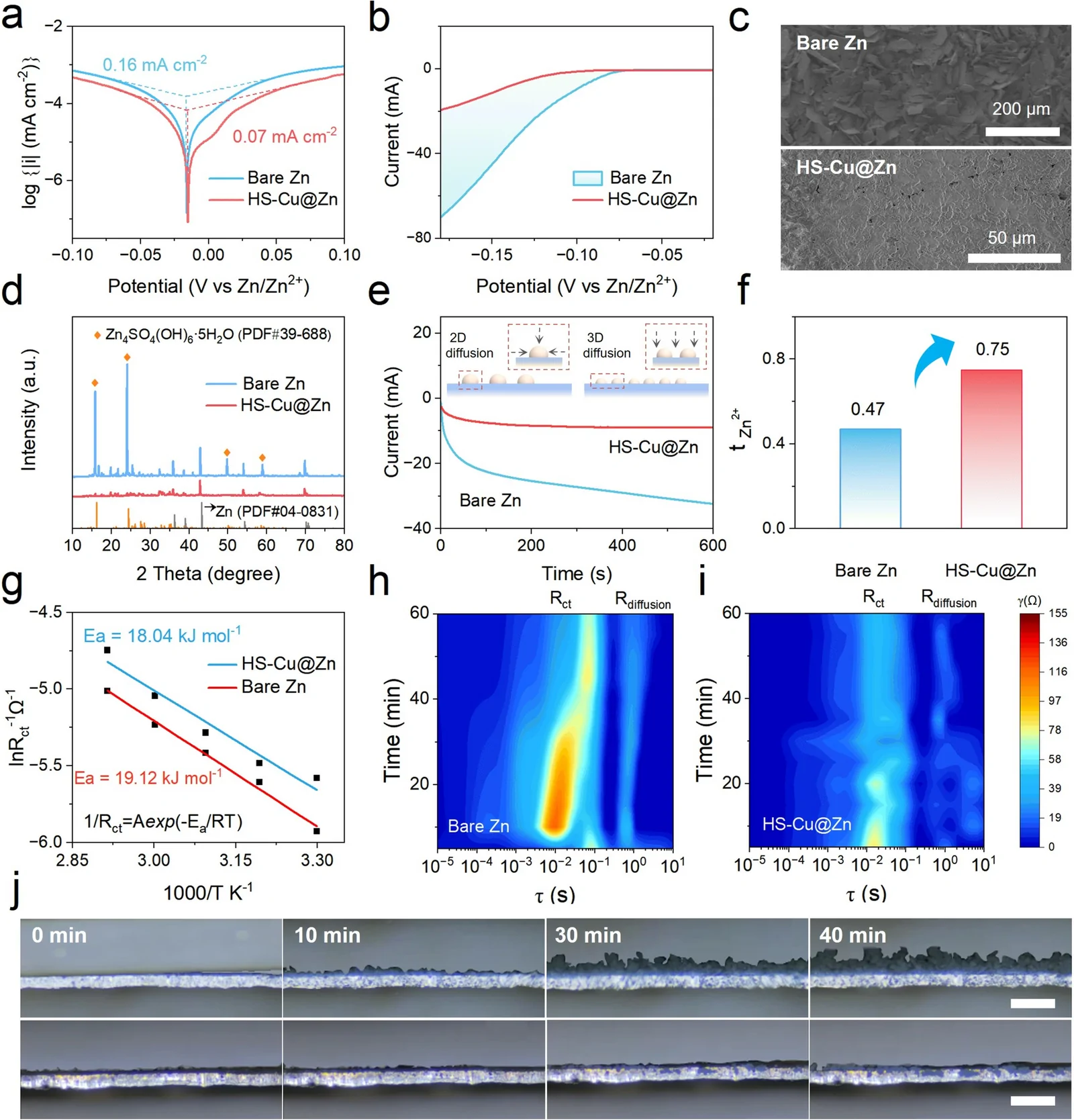
Electrochemical kinetics, corrosion resistance, and Zn deposition behavior of bare Zn and HS-Cu@Zn anodes. **a** Tafel polarization curves and **b** linear sweep voltammetry (LSV) curves of bare Zn and HS-Cu@Zn electrodes recorded at a scan rate of 1 mV s^−^^1^. **c** Surface SEM images and **d** XRD patterns of bare Zn and HS-Cu@Zn electrodes after 50 days of immersion in 2 M ZnSO_4_ electrolyte. **e** Chronoamperometry (CA) curves of different Zn anodes under a − 150 mV overpotential, indicating accelerated 3D Zn diffusion on HS-Cu@Zn. **f** Zn^2+^ transference numbers (*t*_Zn_^2+^) of bare Zn and HS-Cu@Zn symmetric cells. **g** Desolvation activation energies calculated via temperature-dependent EIS and Arrhenius analysis. Distribution of relaxation time (DRT) analysis of symmetric cells based on **h** bare Zn and **i** HS-Cu@Zn during discharge. **j** In situ optical microscopy images capturing Zn deposition dynamics on bare Zn and HS-Cu@Zn anodes at 5 mA cm^−^^2^. Scale bar: 50 μm

To evaluate the electrochemical kinetics and surface stability conferred by the engineered interface, we investigated corrosion resistance, Zn deposition morphology, ion transport properties, and dendrite suppression behavior through a series of electrochemical and microscopic characterizations. Tafel polarization curves show that HS-Cu@Zn anode had a smaller corrosion current density (0.07 mA cm^−2^) and a larger corrosion potential (− 0.015 V) compared to bare zinc (0.16 mA cm^−2^, − 0.016 V) (Fig. 3a). The decrease in corrosion current density indicates a decrease in the corrosion rate and the magnitude of the corrosion potential reflects the degree of difficulty for corrosion to occur [42]. Typically, the reaction kinetics of the HER determines the overall corrosion rate in a zinc-ion battery system. Therefore, the reactivity of the HER was evaluated by linear scanning voltammetry (LSV) testing, and the design of the zincophilic–hydrophobic interface significantly reduced the cathodic current density and increased the HER potential (Fig. 3b). On the contrary, the bare zinc interface accelerated the HER process, making the Zn anode less stable.

Scanning electron microscopy demonstrates the morphological differences between bare Zn and HS-Cu@Zn electrode after 50 days of immersion in 2 M ZnSO_4_ electrolyte. As shown in Fig. 3c, the zinc foils show disorganized and accumulated zinc deposits after immersion, a serious parasitic reaction originating from the corrosion of free water molecules. The alkali by-products generated by the corrosion phenomenon mask the effective reaction sites of the Zn anode, leading to uneven deposition of zinc ions, and the continuous release of OH^−^ during zinc corrosion establishes a concentration gradient to move them toward the cathode, which disrupts the electrolyte equilibrium and leads to a decrease in the capacity of the cathode material [43]. However, the hydrophobic interface constructed by thiol layer prevents water molecules from reacting on the anode, resulting in a flat, dense and smooth morphology. In addition, XRD tests demonstrate that the zinc foil produced a strong signal peak of alkaline zinc sulfate after immersion (Fig. 3d). In contrast, almost no characteristic signals were observed for HS-Cu@Zn. The above results demonstrate the better thermodynamic stability of HS-Cu@Zn compared to bare Zn.

The effect of a zincophilic–hydrophobic interfacial layer on the diffusion and deposition behavior of zinc ions was elucidated using chronoamperometry (CA) test. The bare Zn symmetric cell exhibits a typical two-dimensional diffusion behavior within 600s of current duration (Fig. 3e), and the resulting “tip effect” triggers the formation and growth of dendrites. In contrast, the HS-Cu@Zn exhibits a constant three-dimensional diffusion process with a small and stable current response after a brief two-dimensional diffusion of 150s, suggesting that the modified interfacial layer promotes the uniform nucleation and deposition of zinc. The transference number of Zn^2+^ (*t*_Zn_^2+^) is a critical parameter for enhancing the electrochemical performance of batteries. By analyzing the initial-state impedance (*R*_0_) and steady-state impedance (*R*_s_) of symmetrical cells through I-t curves and Nyquist plots (Fig. S13), the HS-Cu@Zn anode showed a substantially enhanced *t*_Zn_^2+^ value of 0.75, compared to 0.47 for bare Zn (Fig. 3f), demonstrating that the HS-Cu@Zn provides abundant zincophilic sites and high-speed pathways for zinc-ion migration, thereby significantly increasing the Zn^2+^ migration rate. Compared with the bare Zn anode, the integral area of the cyclic voltammetry curve of the HS-Cu@Zn anode in the voltage range of −0.1 to 0.1 V significantly increases (Fig. S14), indicating that the abundant zinc nucleation sites increase the Zn^2+^ concentration at the interface of the HS-Cu@Zn anode, which is conducive to its diffusion [44].

Since Zn^2+^ in aqueous electrolytes usually exists in the hydrated form in the solution of [Zn(H_2_O)_6_]^2+^ and the desolvation process preferentially occurs before Zn deposition, a faster desolvation process not only helps to alleviate the side reactions induced by free water molecules at the Zn anode interface, but also enhances the transport kinetics of Zn^2+^. The activation energy (*E*_a_) was calculated by measuring the electrochemical impedance spectra (EIS) at different temperatures in conjunction with the Arrhenius equation and used to quantitatively describe the desolvation kinetics. The E_a_ of the bare Zn and HS-Cu@Zn anodes is 19.12 and 18.04 kJ mol^−1^, respectively (Figs. 3g and S15), which indicated the stronger desolvation capability of HS-Cu@Zn anode and enhances the electrochemical reaction kinetics. Distribution of relaxation times (DRT) was further used to analyze the transport kinetics of Zn^2+^ at different Zn anode interfaces. Compared to the bare Zn anode (Fig. 3h), both the charge transfer impedance (*R*_ct_) and ion diffusion resistance (*R*_diffusion_) were significantly reduced during zinc deposition at the HS-Cu@Zn anode interface for 60 min at a current density of 2 mA cm^−2^ (Figs. 3i and S16), indicating that the HS-Cu@Zn anode benefits from a “zincophilic -hydrophobic” advantage. As the deposition time increases, the τ value of the bare Zn anode rises, indicating slower interfacial reaction kinetics for zinc ions. Simultaneously, the high electrochemical impedance value of the bare zinc anode indicates hindered charge transfer processes at the interface and significant polarization, thereby reducing stability during zinc deposition. Conversely, the *R*_ct_ parameter of the HS-Cu@Zn anode tends toward stability, suggesting faster desorption response times for zinc ions and more uniform deposition [45–47].

In situ optical microscopy was utilized to record the dynamically evolution of zinc surface morphology during electrolyte–electrode interfacial Zn deposition at different Zn anodes to reveal the role of the zincophilic–hydrophobic interfacial structure in inhibiting zinc dendrite formation (Fig. 3j). After plating for 10 min, obvious granular bumps can be observed on the bare zinc surface, and severe dendrite growth occurs at 40 min of continuous deposition. In contrast, the surface of the HS-Cu@Zn anode maintained a flat deposition morphology due to the rapid desolvation of zinc ions and effective zinc-ion flux regulation.

### Stability and Reversibility of HS-Cu@Zn Anodes

**Fig. 4:**
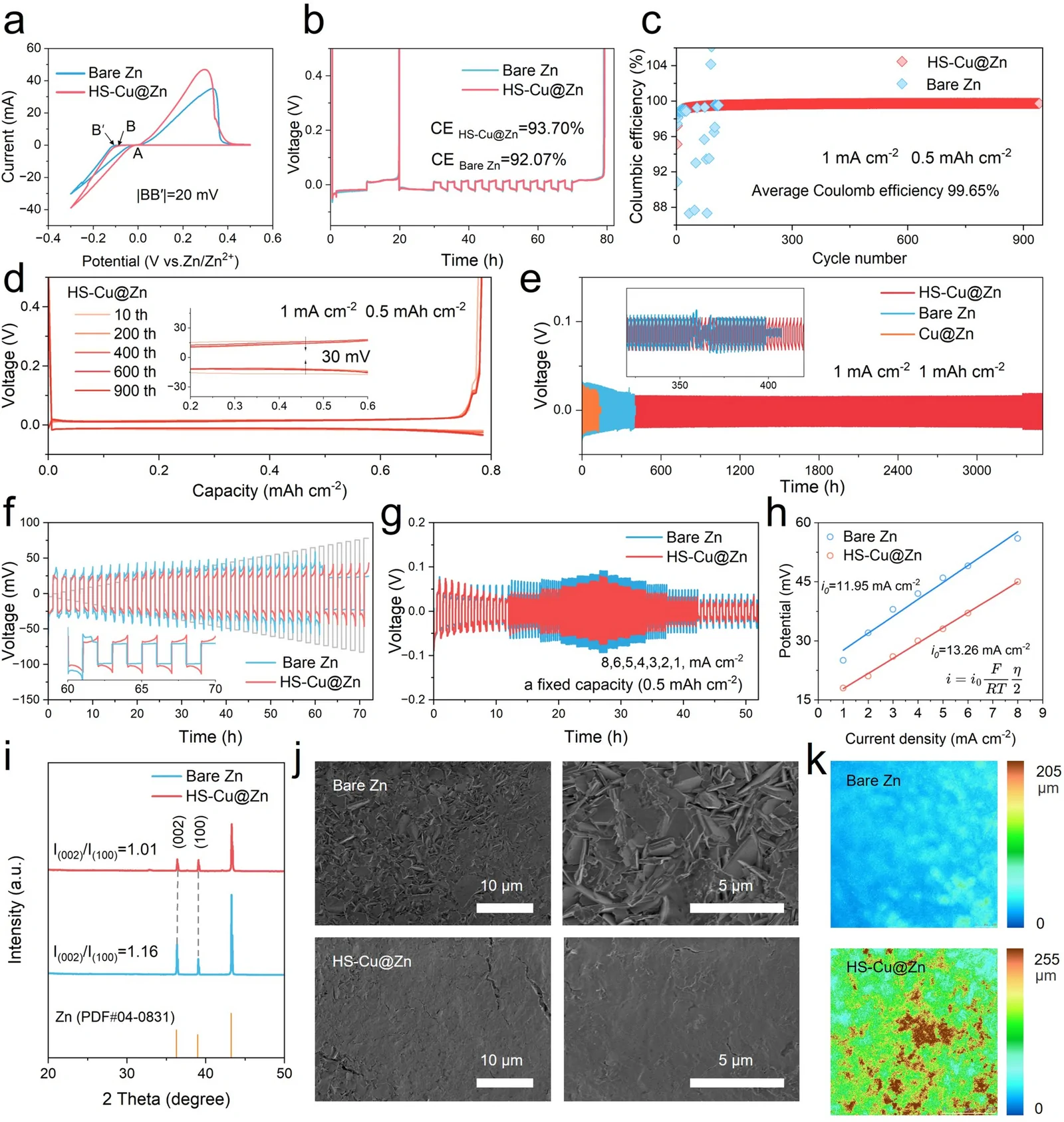
Electrochemical performance of the HS-Cu@Zn and bare Zn anode in symmetric cells. **a** CV curves of Zn||Ti half-cells. **b** CE of bare Zn and HS-Cu@Zn anodes. **c** Coulombic efficiency of Zn plating/stripping in Cu||Zn and Cu||HS-Cu@Zn asymmetric cells at a current density 1 mA cm^−2^ with a capacity of 0.5 mAh cm^−2^. **d** Polarization curves of Cu||HS-Cu@Zn cells at the 10th, 200th, 400th, 600th, and 900th cycles, respectively. **e** Cycling performance of bare Zn, Cu@Zn, and HS-Cu@Zn symmetric cells at 1 mA cm^−2^ for 1 mAh cm^−2^. **f** Potential evolution of symmetric cells at step-increased current densities. **g** Rate performance of HS-Cu@Zn and bare Zn symmetric cells at various current densities and **h** corresponding exchange current density. **i** XRD analysis and **j** corresponding SEM and **k** CLSM 2D height images of the bare Zn and HS-Cu@Zn anodes after 50 cycles at 1 mA cm^−2^, 1 mAh cm^−2^

The long-term electrochemical reversibility of the HS-Cu@Zn anode was evaluated through a comprehensive set of half-cell and symmetric cell measurements. Cyclic voltammetry (CV) of Zn||Ti asymmetric cells revealed that HS-Cu@Zn exhibited stronger current response and lower overpotentials than bare Zn anode (Fig. 4a), indicating improved reaction kinetics and plating/stripping reversibility. The utilization and reversibility of Zn were quantified using a “reservoir Zn||Cu half-cell” protocol. The HS-Cu@Zn anode achieved an average CE of 93.7% and maintained highly stable voltage profiles over prolonged cycling, while the relatively low CE of the bare Zn||Cu highlighted its susceptibility to parasitic side reactions (Fig. 4b). The CE was further tested using Zn||Cu half-cells to evaluate the reversibility of Zn plating/stripping. The cell with HS-Cu@Zn anode could reach up to 900 stable cycles with CE of 99.65%, while the cell assembled with bare Zn anode failed to operate after less than 150 cycles (Fig. 4c), indicating that the interfacial layer of “zincophilic–hydrophobic” interface ensures a high reversibility of Zn plating/stripping. In addition, the polarization curves of the HS-Cu@Zn anode after 900 cycles were characterized by a low voltage gap (≈ 30 mV) and almost overlapping, evidencing that the corrosion phenomenon was suppressed (Fig. 4d). In contrast, the capacity curves of the bare Zn anodes showed an unstable trend, indicating that the Zn||Cu cell began to experience rapid and irreversible zinc deposition after 40 cycles, resulting in a drastic change in CE and cell failure (Fig. S17). These results suggest that the bifunctional interfacial layer of zincophilic and hydrophobic formed by Cu–S covalent bonds facilitated charge transfer of Zn^2+^, constructed abundant ion transport channels for Zn^2+^, and significantly reduced the decomposition of water molecules on the anode surface.

Due to its enhancement of kinetic stability and inhibition of side reactions, the symmetric cell assembled with HS-Cu@Zn delivered exceptional cycling stability over 3500h at 1 mA cm^−2^ and 1 mAh cm^−2^, significantly outperforming bare Zn (407 h) and Cu@Zn (145 h) (Fig. 4e). Compared to bare Zn anodes, the Zn–Cu interface decreases the stability of the anode due to the enhanced hydrophilicity. Even under a high current density of 10 mA cm^−2^, the HS-Cu@Zn anode maintained stable operation for 500 h, whereas bare Zn failed after only 130 h (Fig. S18). Furthermore, the rate capability of the Zn anode was evaluated by gradually increasing the current density. As depicted in Fig. 4f, the cell with the HS-Cu@Zn anode presented stable voltage curves within the current density range of 0.25–9.0 mA cm^−2^. For the bare Zn anode, the voltage curve becomes abnormal and short-circuited when the current density exceeds 4.0 mA cm^−2^. The results suggest that the “zincophilic–hydrophobic” interface possesses favorable zinc-ion conductivity and interface charge transfer ability. This particular charge transfer can be attributed to the alteration of charge distribution on the anode surface due to the establishment of Cu nanolayers [48], which reduces the electron cloud density of the Zn atoms. The rate performance of symmetric cells assembled with different Zn anodes at current densities of 1–8 mA cm^−2^ was studied by fixing a capacity of 0.5 mAh cm^−2^ (Figs. 4g and S19). It can be seen that the HS-Cu@Zn cells have stable and small voltage hysteresis at current densities of 1, 2, 3, 4, 5, 6, and 8 mA cm^−2^, demonstrating excellent interfacial charge transfer capability. In contrast, despite the bare Zn cell maintaining stable operation throughout the test, as the deposition capacity and rate increase, a greater number of Zn^2+^ undergo charge transfer at the interface. However, the bare Zn electrolyte interface lacks the capability to accommodate these increased charge transfers, resulting in severe voltage hysteresis, which will lead to a decrease in the Zn utilization. The exchange current density associated with the Zn electrodeposition process was obtained from the Butler–Volmer equation approximation. The fitting results showed that HS-Cu@Zn (13.26 mA cm^−2^) exhibited a higher exchange current density than bare Zn (11.95 mA cm^−2^), demonstrating the beneficial effect of the “zincophilic–hydrophobic” layer on Zn deposition kinetics (Fig. 4h).

In addition, differences in zinc deposition behavior on different Zn anodes were investigated. As shown in Fig. 4i, XRD pattern of zinc plating on Zn||Zn symmetric cells after 50 cycles of 1 and 1 mAh cm^−2^ was tested. The (002)/(100) peak intensity ratio during Zn deposition changes from 1.16 (bare Zn) to 1.01 (HS-Cu@Zn), and this decrease indicates that the “zincophilic-hydrophobic” interface favors preferential deposition of Zn^2+^ on the (100) plane. It is noteworthy that in the hexagonal close-packed (hcp) structure of zinc, the (100) plane is oriented perpendicular to the anode surface [49]. Therefore, when the (100) plane is densely deposited, a flat zinc metal anode is formed. Surface morphology analysis by SEM revealed the surface of the bare Zn anode after cycling was rough and inhomogeneous (Fig. 4j), and the staggered direction of Zn^2+^ deposition led to the significant growth of dendrites. In contrast, the HS-Cu@Zn anode after recycling had a dense and uniform deposition layer. Similarly, the symmetric cell shows a similar Zn anode morphology after 20 cycles (Fig. S20), indicating that the effect of water molecules on the reversibility of the Zn anode is not negligible even during the initial cycling of the cell. In addition, confocal laser scanning microscopy (CLSM) images also showed bare Zn after cycling with significant height changes (Fig. 4k), demonstrating the rampant growth of dendrites. The HS-Cu@Zn anode surface remained flat. Simultaneously, XPS characterization verifies that the stability of thiol groups on the HS-Cu@Zn surface remains unimpaired after long-term electrochemical cycling; the S 2*p* binding energy is consistent with that of the pristine HS-Cu@Zn, and no additional characteristic peaks are detected in the test spectra (Fig. S21). The above results indicate that the zincophilic–hydrophobic interface is favorable for inhibiting the generation of by-products and increasing the reversibility of zinc-ion plating/stripping.

### Electrochemical Performance of ZnVO-Based Full Cells with HS-Cu@Zn Anodes

**Fig. 5:**
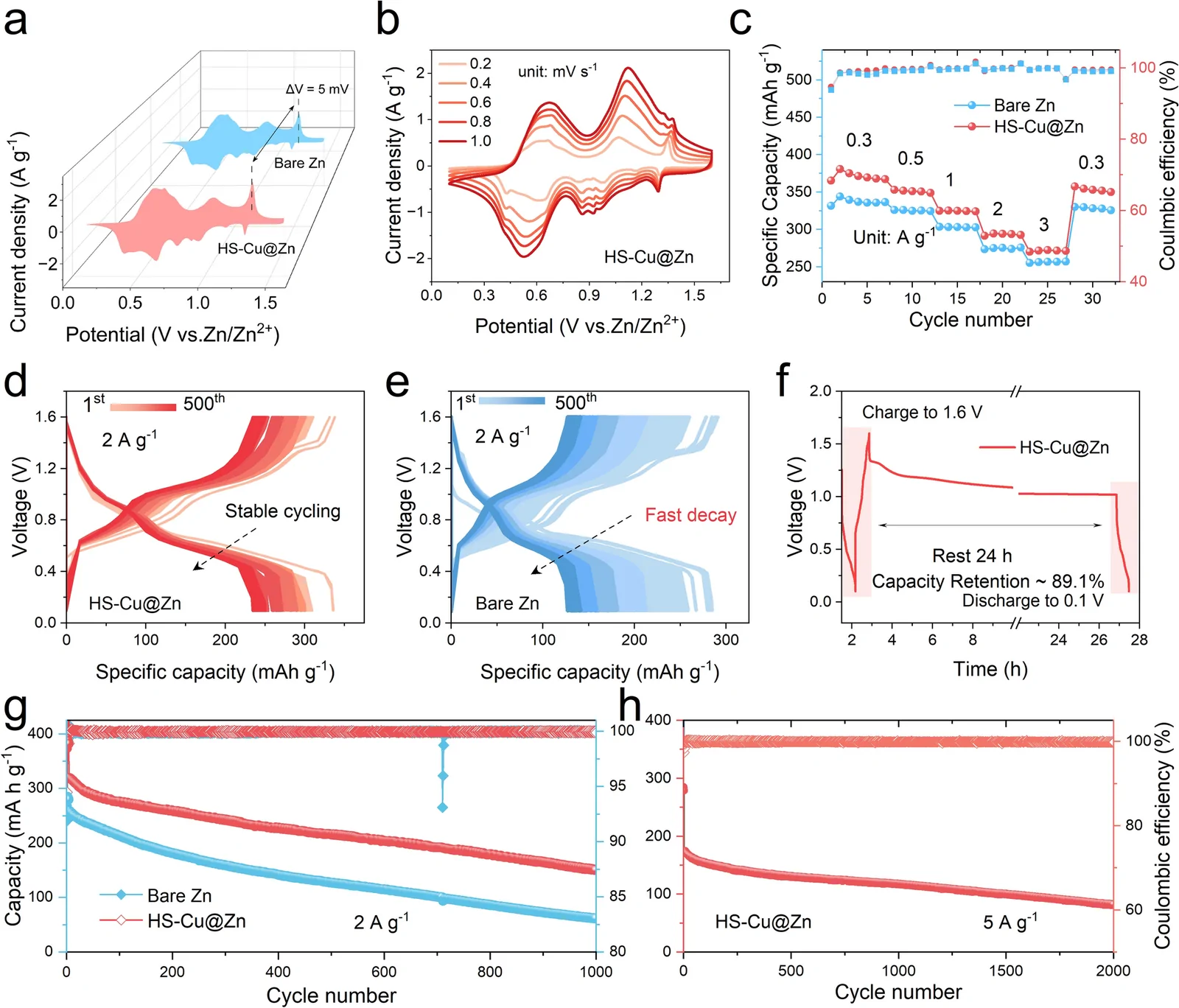
Electrochemical performance of ZnVO-based full cells employing bare Zn and HS-Cu@Zn anodes. **a** CV curves of ZnVO||bare Zn and ZnVO||HS-Cu@Zn full cells at a scan rate of 0.2 mV s^−^^1^. **b** CV profiles of ZnVO||HS-Cu@Zn full cell under various scan rates. **c** Rate performance of full cells tested in 2 M ZnSO_4_ + 0.1 M MnSO_4_ electrolyte. Voltage profiles of **d** ZnVO||HS-Cu@Zn and **e** ZnVO||bare Zn full cells at 2 A g^−^^1^ over 500 cycles. **f** Capacity retention of ZnVO||HS-Cu@Zn cell after resting for 24h following charge to 1.6 V and discharge to 0.1 V. **g** Long-term cycling performance comparison of ZnVO||bare Zn and ZnVO||HS-Cu@Zn cells at 2 A g^−^^1^ after initial activation cycles at 0.5 A g^−^^1^. **h** High-rate cycling stability of ZnVO||HS-Cu@Zn full cell at 5 A g^−^^1^ with excellent capacity retention and coulombic efficiency

In order to explore the potential of HS-Cu@Zn anode for practical applications, full cells with HS-Cu@Zn as anode and ZnVO as cathode were assembled. CV curves of the full cells using HS-Cu@Zn and bare Zn anodes were tested at 0.2 mV s^−1^ and showed similar redox peaks (Fig. 5a), but the voltage polarization during cycling was lower for the full cell using HS-Cu@Zn anodes, suggesting that it favors Zn^2+^/Zn reaction kinetics. Moreover, CV profiles at different scan rates (Fig. 5b) for ZnVO||HS-Cu@Zn reveal minimal peak shifting and broadening, suggesting robust rate capability and charge transfer. The EIS results also show that the R_ct_ of the ZnVO||HS-Cu@Zn full cell was smaller, which confirms that the modified interface suppresses the side reactions and increases the fast redox kinetics of Zn ions (Fig. S22).

The enhanced reaction kinetics of the modification of the Zn anode interface also contributes to the improvement of the rate performance of the full cell. Figure 5c demonstrates that the discharge specific capacities of the ZnVO||HS-Cu@Zn full cell were 450, 430, 404, 373, and 351 mAh g^−1^ at current densities of 0.3, 0.5, 1, 2, and 3 A g^−1^, respectively. In contrast, full cells assembled with bare Zn anodes exhibited lower capacity under the same conditions. Long-term cycling performance at 2 A g^−1^ revealed rapid capacity fading in the ZnVO||bare Zn cell, as evidenced by the polarization curves for 500 cycles (Fig. 5d). In contrast, the full cell assembled with the HS-Cu@Zn anode showed stable cycling performance (Fig. 5e).

In addition, self-discharge performance is one of the characteristics to evaluate the durability of the battery during the storage period. Figures 5f and S23 show that by monitoring the voltage change of the full cell after 24h of storage, it was found that the ZnVO||HS-Cu@Zn possessed a higher capacity retention rate (89.1%), compared with 86% for the bare Zn-assembled full cell, which further clarifies the effectiveness of the modified interface in inhibiting the side reactions. The cycling stability of the cells was evaluated at 2 A g^−1^ after 5 cycles at a low current density of 0.5 A g^−1^ (Fig. 5g). The capacity of the assembled ZnVO||HS-Cu@Zn cell after 1000 cycles was 150 mA h g^−1^. In comparison, the capacity of the ZnVO|| bare Zn cell after cycling was only 61.5 mA h g^−1^ with a capacity retention rate of 23.8%. In addition, Fig. 5h demonstrates that the HS-Cu@Zn cell also had excellent cycling stability at 5 A g^−1^ with an average coulombic efficiency of 99.8%. This indicates that HS-Cu@Zn has better interfacial stability than bare Zn.

## Conclusions

A simple and highly efficient synergistic interfacial engineering strategy was used to realize a “zincophilic–hydrophobic” Zn anode interface, thus reducing the desolvation energy of zinc ions and achieving higher ion transference number (0.75). Specifically, the loosely rough surface derived from Cu nanorod arrays provides ordered zinc migration channels and serves as a conductive substrate, regulating the nucleation sites of Zn^2+^ ions and promoting their uniform deposition. Meanwhile, the surface hydrophobicity imparted by terminal functional groups of HS molecules effectively minimizes water molecule concentration at the anode interface, ensuring enhanced stability and reversibility of the Zn anode. Therefore, the symmetric battery assembled with HS-Cu@Zn exhibits stable and consistent performance for 3500h at 1 mA cm^−2^. Even under a high current density of 10 mA cm^−2^, it maintained a cycling stability of 500h. The Zn||Cu half-cell maintain a CE of 99.56% after 900 cycles. The assembled ZnVO||HS-Cu@Zn full cell had an average CE of 99.8% at 5 A g^−1^, exhibiting an excellent cycling stability.

## Supplementary Information

Below is the link to the electronic supplementary material.

Supplementary file1 (DOCX 10513 KB)
