# Supplementary material for: Zincophilic–Hydrophobic Interface Design for Dendrite-Free Aqueous Zinc-Ion Batteries
Source: Nanomicro Lett. 2026 Apr 9;18:324. doi: 10.1007/s40820-026-02153-4 (PMC13065845; doi:10.1007/s40820-026-02153-4)
Supplement: Supplementary file 1 — Supplementary file1 (DOCX 10513 KB) [file 40820_2026_2153_MOESM1_ESM.docx]

Supporting Information for

**Zincophilic-Hydrophobic Interface Design for Dendrite-Free Aqueous Zinc-Ion Batteries**

Yinfeng Guo^1#^, Yuxiang Xu^1, 2#^, Yaduo Jia^3^, Xiaoqing Zhu^1^, Tao Zhang^1^, Jia Zhang^1^, Changyong (Chase) Cao^4,5^, Qilin Gu^6^, Guiyin Xu^1^*

^1^State Key Laboratory of Advanced Fiber Materials, College of Materials Science and Engineering, Donghua University, Shanghai 201620, P. R. China

^2^Ancan Technology LTD, Jiangsu 214400, P. R. China

^3^Tianjin Key Laboratory of Materials Laminating Fabrication and Interface Control Technology, School of Materials Science and Engineering, Hebei University of Technology, Tianjin 300130, P. R. China

^4^Department of Mechanical and Aerospace Engineering, Case Western Reserve University, Cleveland, OH 44106, USA

^5^Advanced Platform Technology (APT) Center, Louis Stokes Cleveland VA Medical Center, Cleveland, OH 44106, USA

^6^NJTECH University Suzhou Future Membrane Technology Innovation Center, Suzhou 215100, P. R. China

^#^Yinfeng Guo and Yuxiang Xu contributed equally to this work

*Corresponding author. E-mail: [xuguiyin@dhu.edu.cn](mailto:xuguiyin@dhu.edu.cn) (Guiyin Xu)

**S1 Material Characterizations**

Scanning electron microscopy (SEM; HITACHI SU8010, Japan) was used to examine the surface morphology. X-ray diffraction (XRD; PANalytical X’Pert Pro, Netherlands) was employed to characterize crystal structures, using Cu Kα radiation (λ = 1.5406 Å). X-ray photoelectron spectroscopy (XPS; Thermo Scientific ESCALAB 250Xi, UK) was used to analyze elemental composition and valence states. Confocal laser scanning microscopy (CLSM; Keyence VK-X1100) provided 2D height profiles. Fourier-transform infrared spectroscopy (FTIR; Bruker INVENIO-R) was used to characterize chemical bonding. Static water contact angles were measured using a Nikon D90 camera.

**S2 Electrochemical measurements**

The cyclic voltammetry (CV), linear sweep voltammetry (LSV), electrochemical impedance spectroscopy (EIS), and Tafel were evaluated using CHI760E electrochemical workstation. EIS spectra were acquired within frequency from 10^−5^ to 10^−2^ Hz using a 5 mV AC amplitude. Potentiodynamic polarization tests were carried out at a scan rate of 1 mV s^−1^. The differential capacitance curve is calculated using the formula C=−(ωZ_im_)^−1^, where C represents the differential capacitance, ω denotes the angular frequency, and Z_im_ is the imaginary part of the impedance. To eliminate interference from Zn^2^⁺ deposition behavior on the test results, this experiment employs Na_2_SO_4_ solution as the test solution. Cells performance was tested on NEWARE and LAND battery testing systems. To investigate the electrochemical stripping/plating behavior and cycling stability of the bare Zn and HS-Cu@Zn anode, the symmetric cells were assembled in CR2025 coin-type cells using a glass fiber separator (Whatman, GF/A) and 2 M ZnSO_4_ aqueous electrolyte. Full cells were assembled with bare Zn or HS-Cu@Zn as the anode and ZnVO as the cathode.

**S3 Computational Methods**

DFT simulations were performed to gain molecular-level insights into surface interactions, charge redistribution, and Zn^2^⁺ desolvation dynamics at the engineered electrode interface. All geometry optimizations of small molecules, including 1-dodecanethiol and H_2_O, were conducted using the ORCA 6.0.1 software suite. The B3LYP functional was employed along with the def2-SVP basis set and Grimme's D4 dispersion correction for structure optimization. Single-point energy calculations utilized the higher-accuracy def2-TZVP basis set to improve electronic property accuracy. ESP maps, highest occupied molecular orbital (HOMO) and lowest unoccupied molecular orbital (LUMO) energies, and differential charge densities were calculated using Multiwfn and visualized using VMD software.

To assess surface binding characteristics and Zn atom adsorption energetics, plane-wave DFT calculations were carried out using the Vienna Ab-initio Simulation Package (VASP). The exchange-correlation interactions were treated with the Perdew–Burke–Ernzerhof (PBE) functional within the generalized gradient approximation (GGA). The Zn(001) and Zn(100) surfaces were modeled as symmetric periodic slabs consisting of 4×4 and 5×2 supercells, respectively, separated by sufficient vacuum spacing to prevent interlayer interactions. The hybrid HS-Cu@Zn surfaces were constructed by depositing a layer of Cu and adsorbing stearate anions onto the Zn slab.

The Brillouin zone was sampled using a Gamma-centered 3×3×1 k-point mesh, and the electronic energy convergence criterion was set to 10⁻⁵ eV. Adsorption energy ($\Delta E_{ads}$) of species was calculated using the following expression:

$$\Delta E_{\text{ads}}=E_{\text{total}}-E_{\text{slab}}-E_{\text{ad}},$$

where $E_{\text{total}}$ denotes the total energy of the system with adsorbate, and $E_{\text{slab}}$ and $E_{\text{ad}}$ represent the energies of the bare surface and isolated adsorbate, respectively. These calculations revealed critical insights into the improved Zn affinity and water-repelling capabilities of the HS-Cu@Zn surface, consistent with experimental observations.

**Supplementary Figures and Tables**


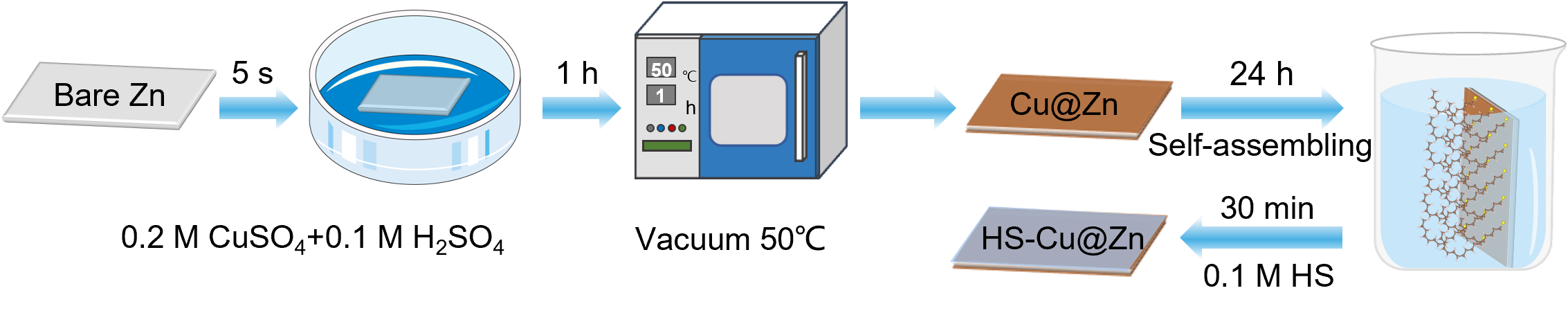


**Fig. S1** Schematic diagram of the preparation process of Cu@Zn and HS-Cu@Zn anode


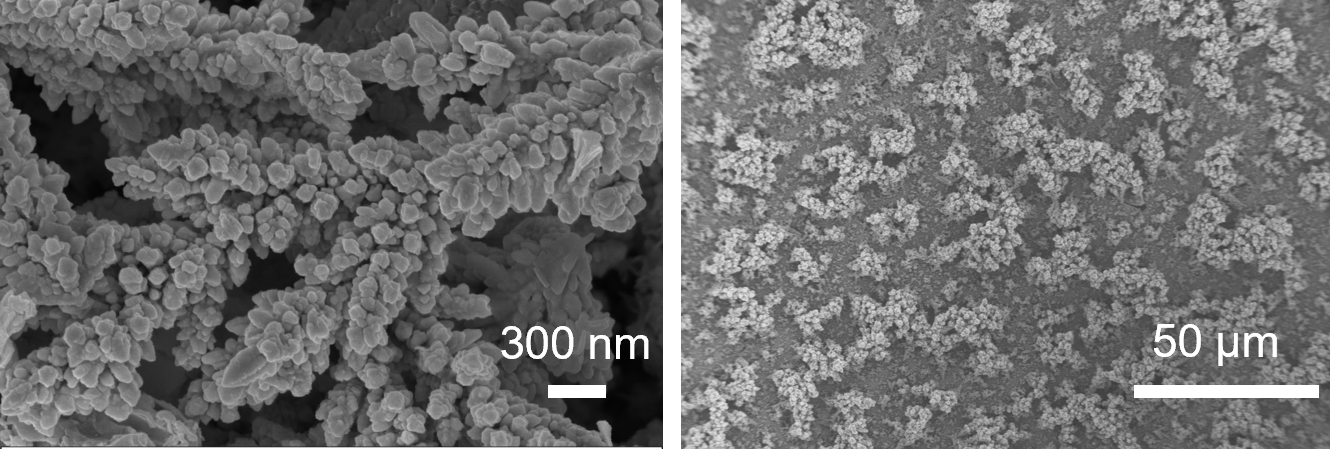


**Fig. S2** SEM images of Cu@Zn anode


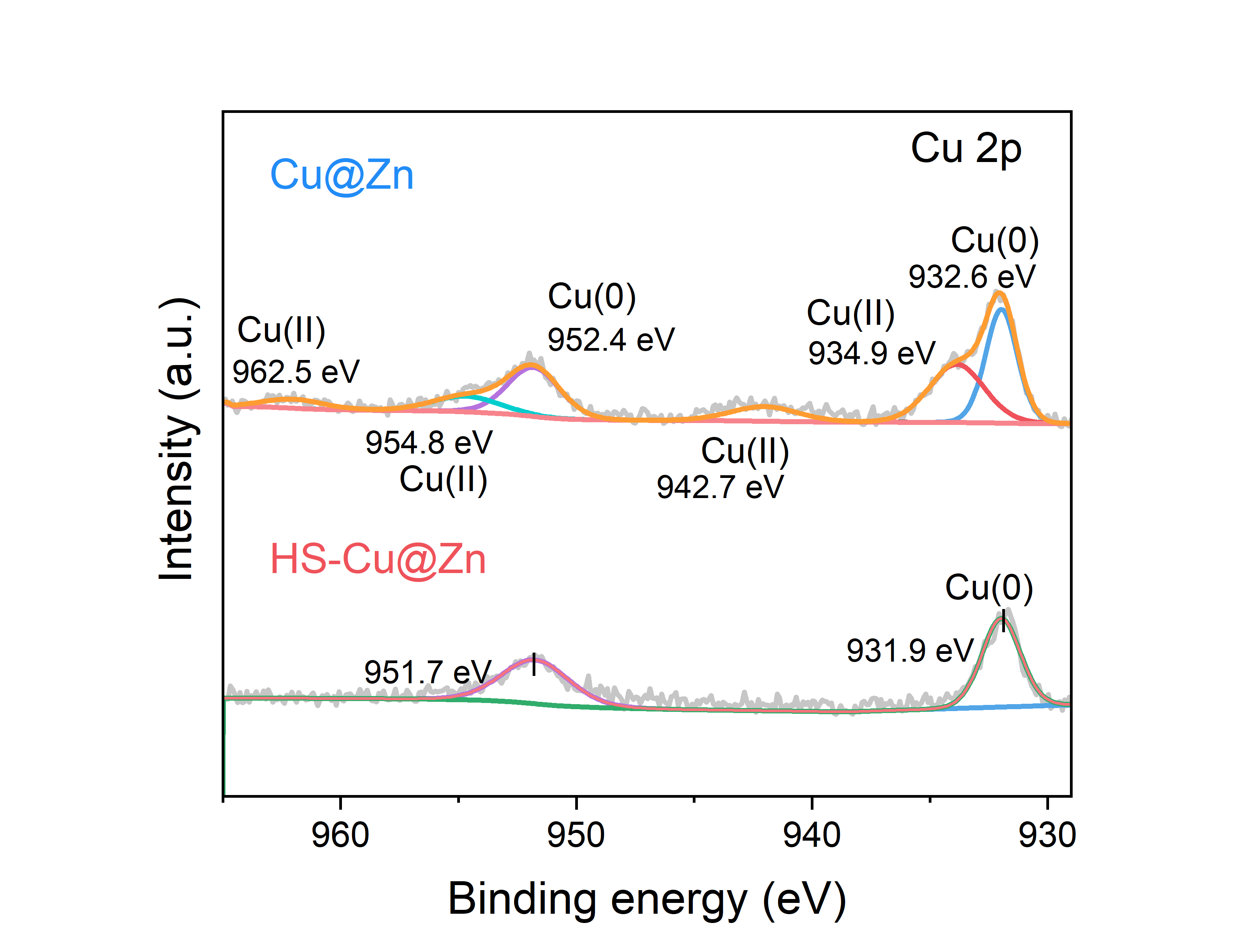


**Fig. S3** XPS high-resolution spectra of Cu 2p for Cu@Zn and HS-Cu@Zn


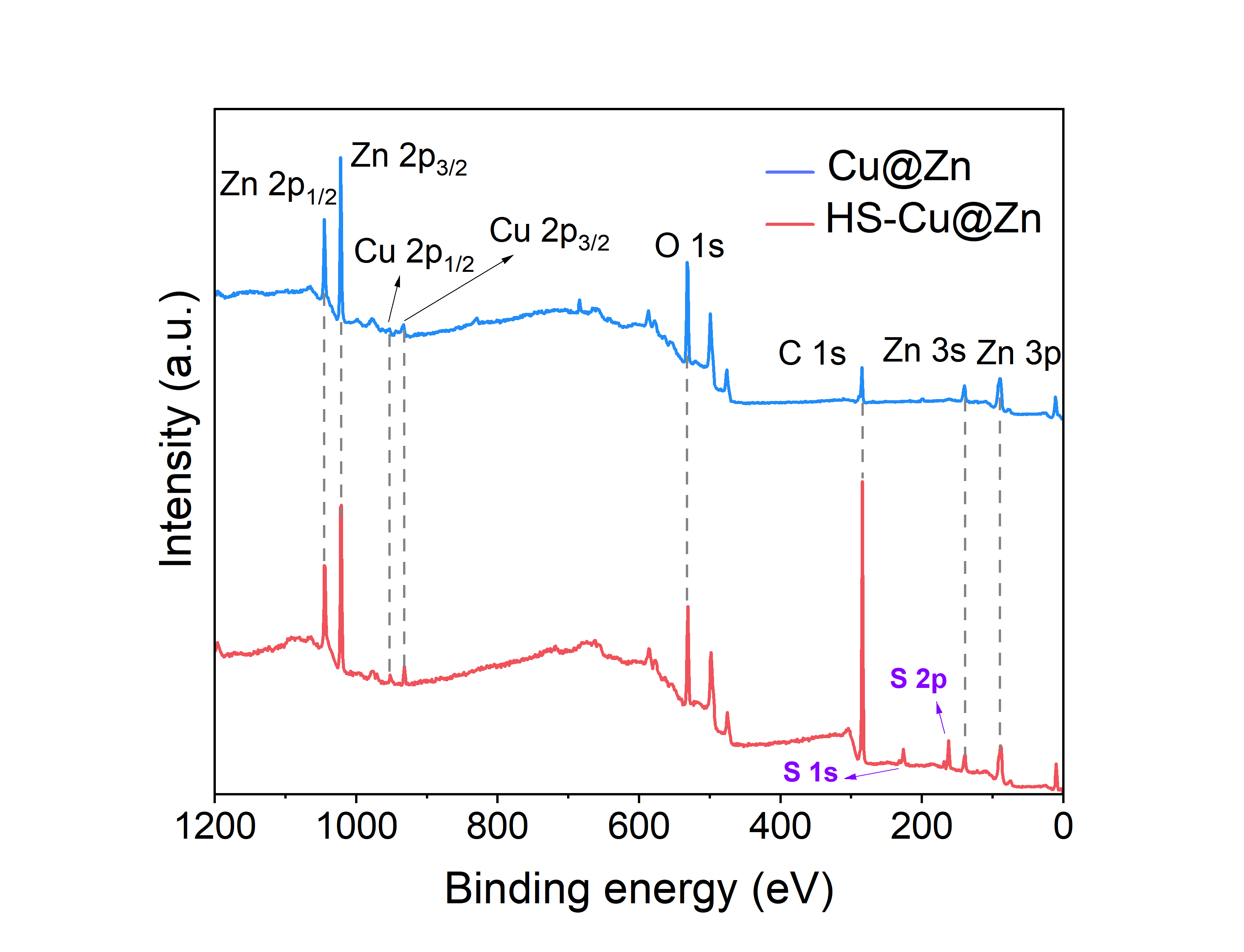


**Fig. S4** Survey XPS spectra for Cu@Zn and HS-Cu@Zn


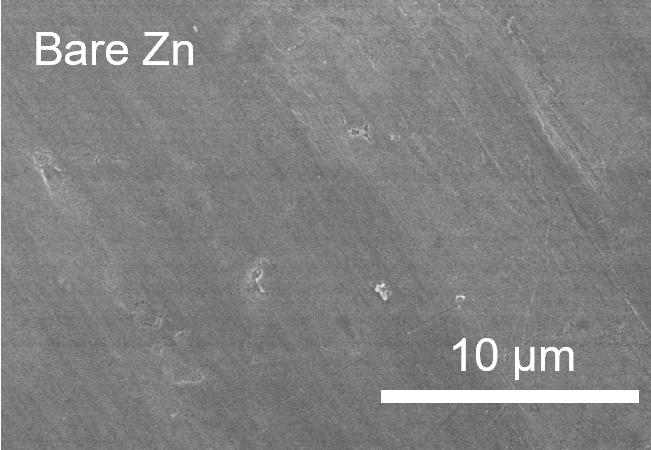


**Fig. S5** SEM image of bare Zn anode


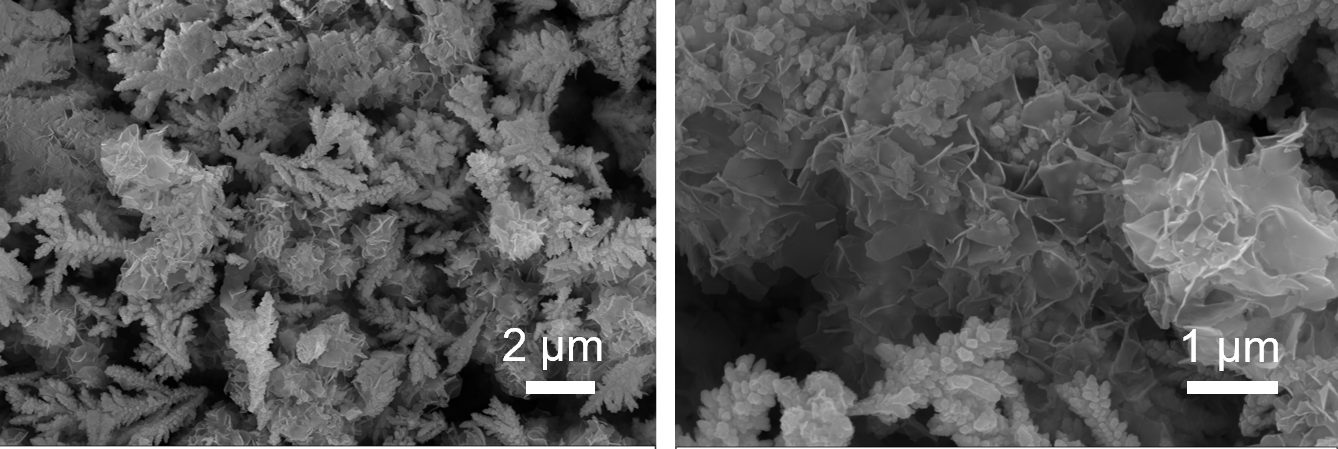


**Fig. S6** SEM image of HS-Cu@Zn anode


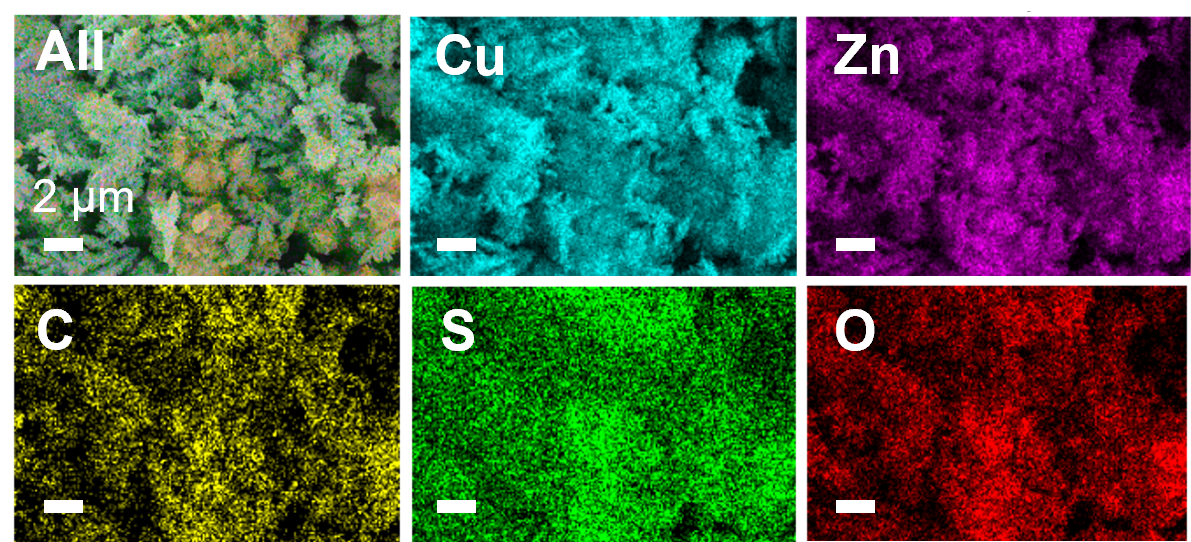


**Fig. S7** SEM image and the corresponding elemental mappings of the HS-Cu@Zn anode


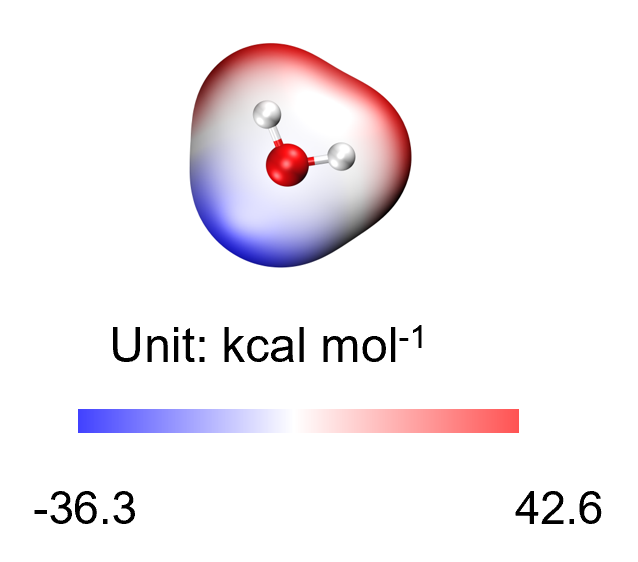


**Fig. S8** The ESP of the H_2_O





**Fig. S9** CV curves of (**a**) HS-Cu@Zn and (**b**)Bare Zn symmetrical cells at different scan rates; EDL capacitance of (**c**) HS-Cu@Zn and (**d**)Bare Zn symmetric cells


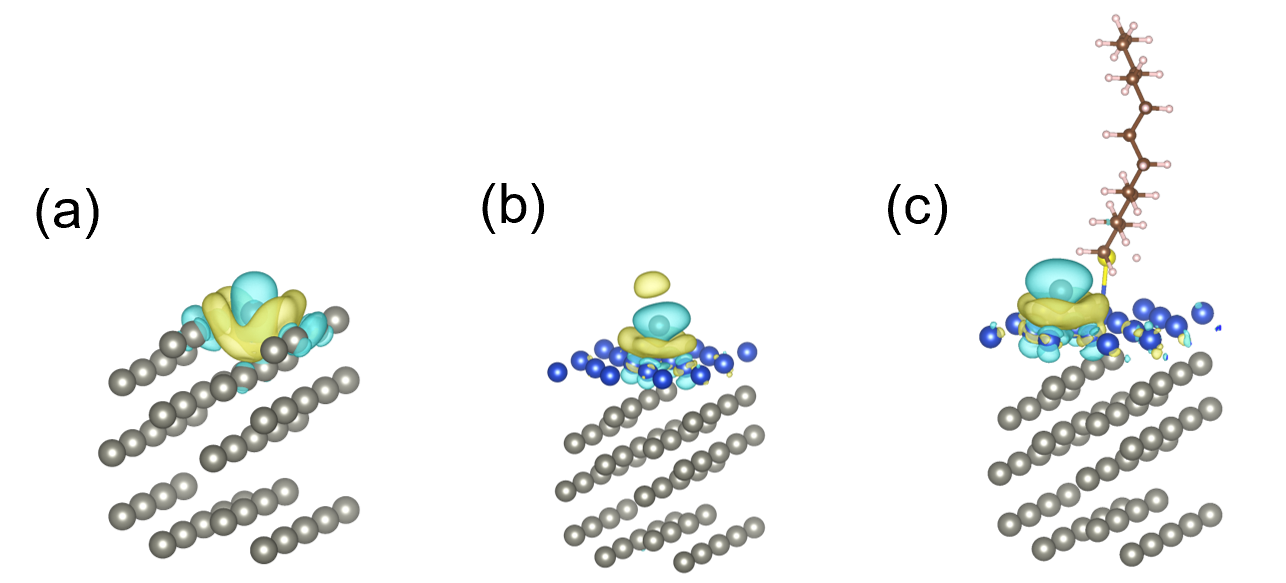


**Fig. S10** Schemes showing Zn atom adsorbed on the surfaces of (**a**) Zn, (**b**) Zn@Cu and (**c**) HS-Cu@Zn


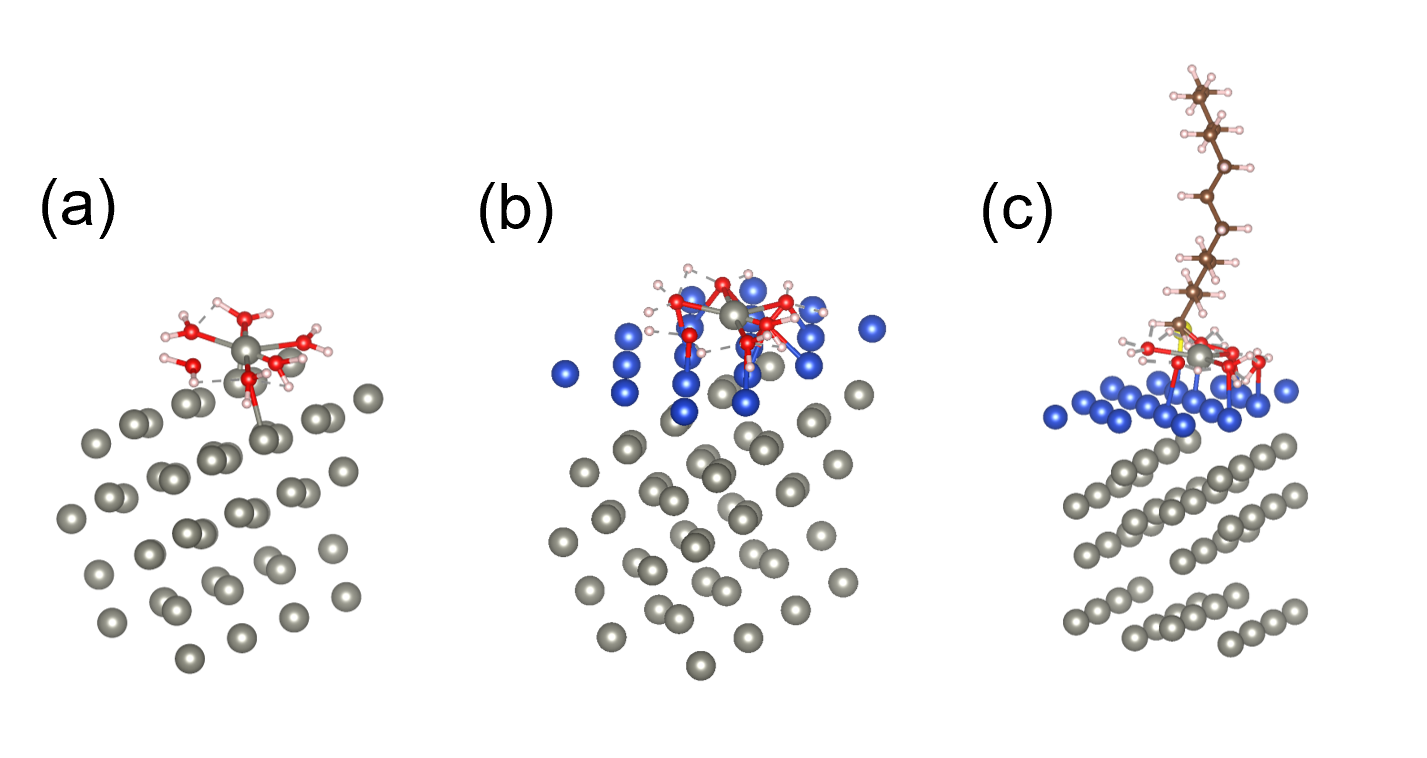

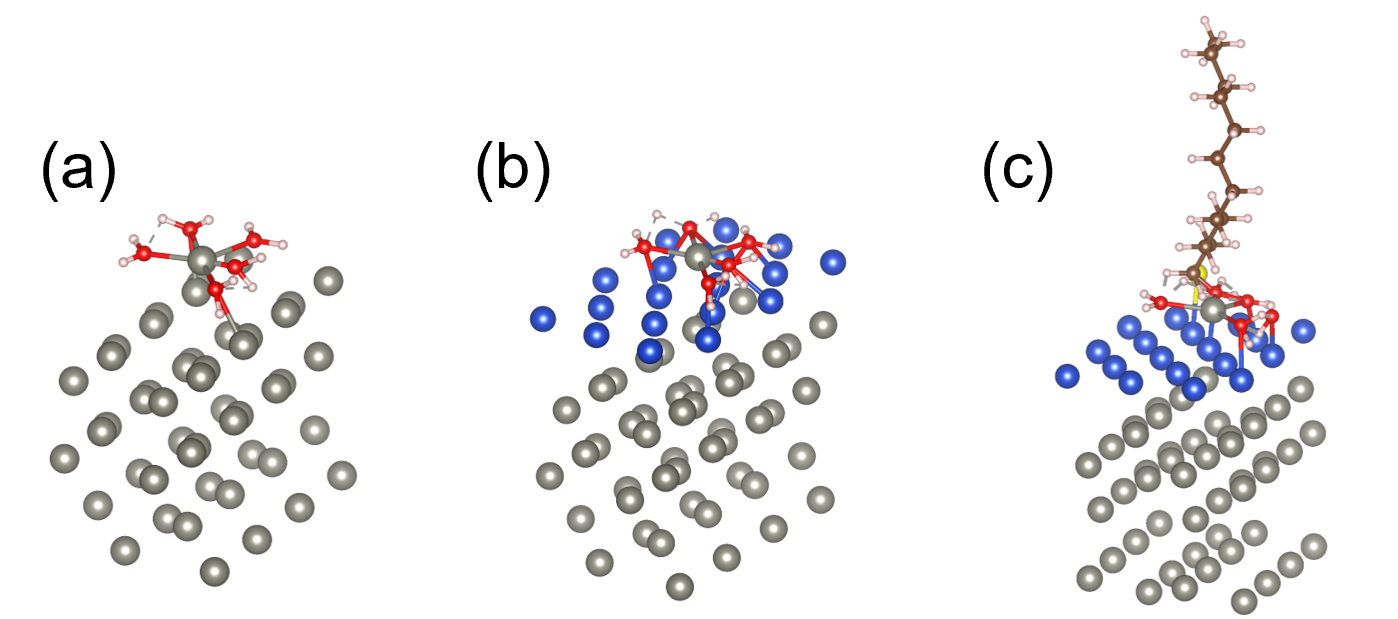


**Fig. S11** Schemes showing the removal of a single water molecule from the solvated structure of zinc ions on the surface of (**a**) Zn, (**b**) Zn@Cu and (**c**) HS-Cu@Zn.


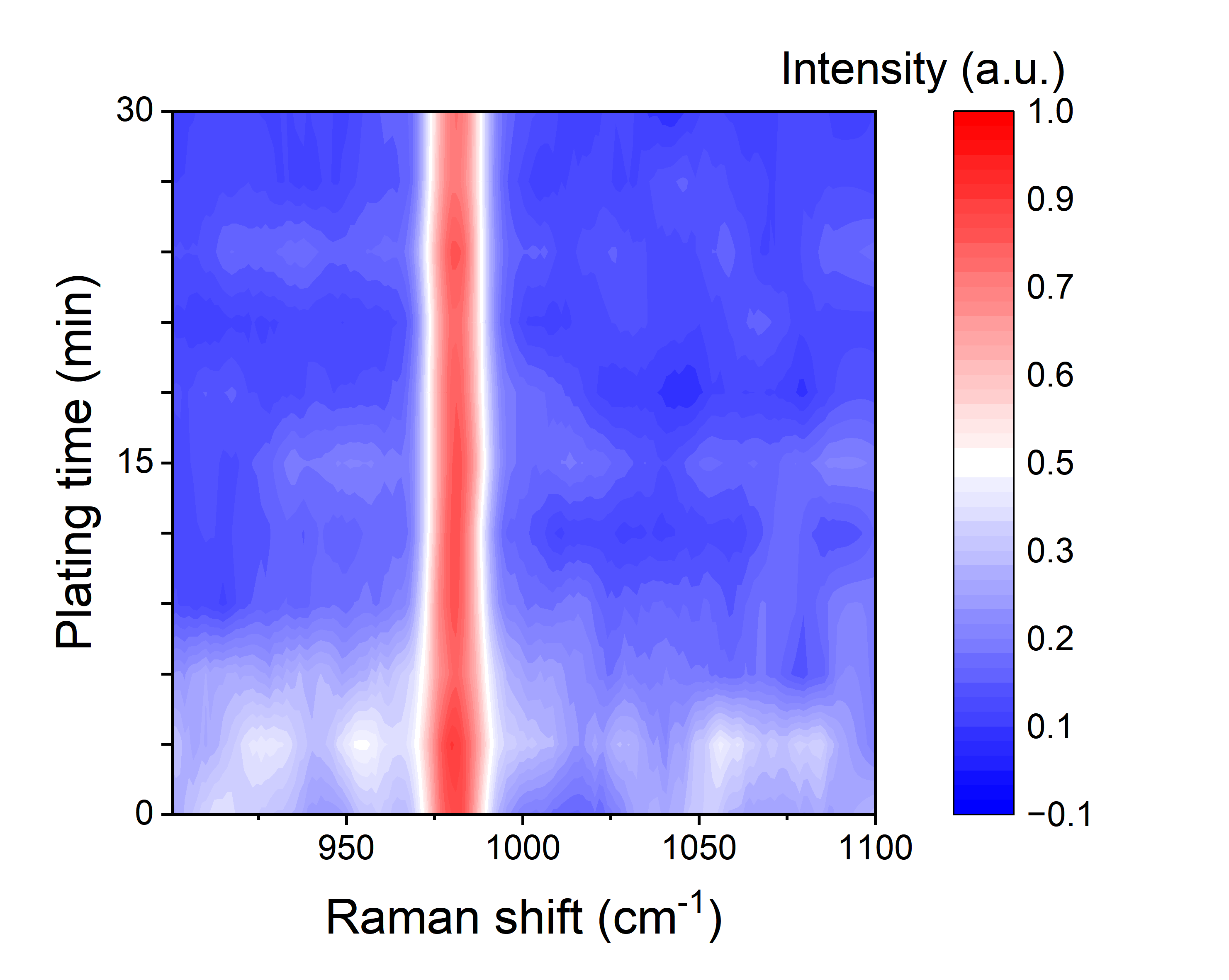


**Fig. S12** In situ Raman spectra of Bare Zn anode surfaces

**Fig. S13** Current-time curves and corresponding Nyquist plots (inset) of (**a**) bare Zn and (**b**) HS-Cu@Zn before and after polarization at 20 mV for the *t*_Zn_^2+^ measurement


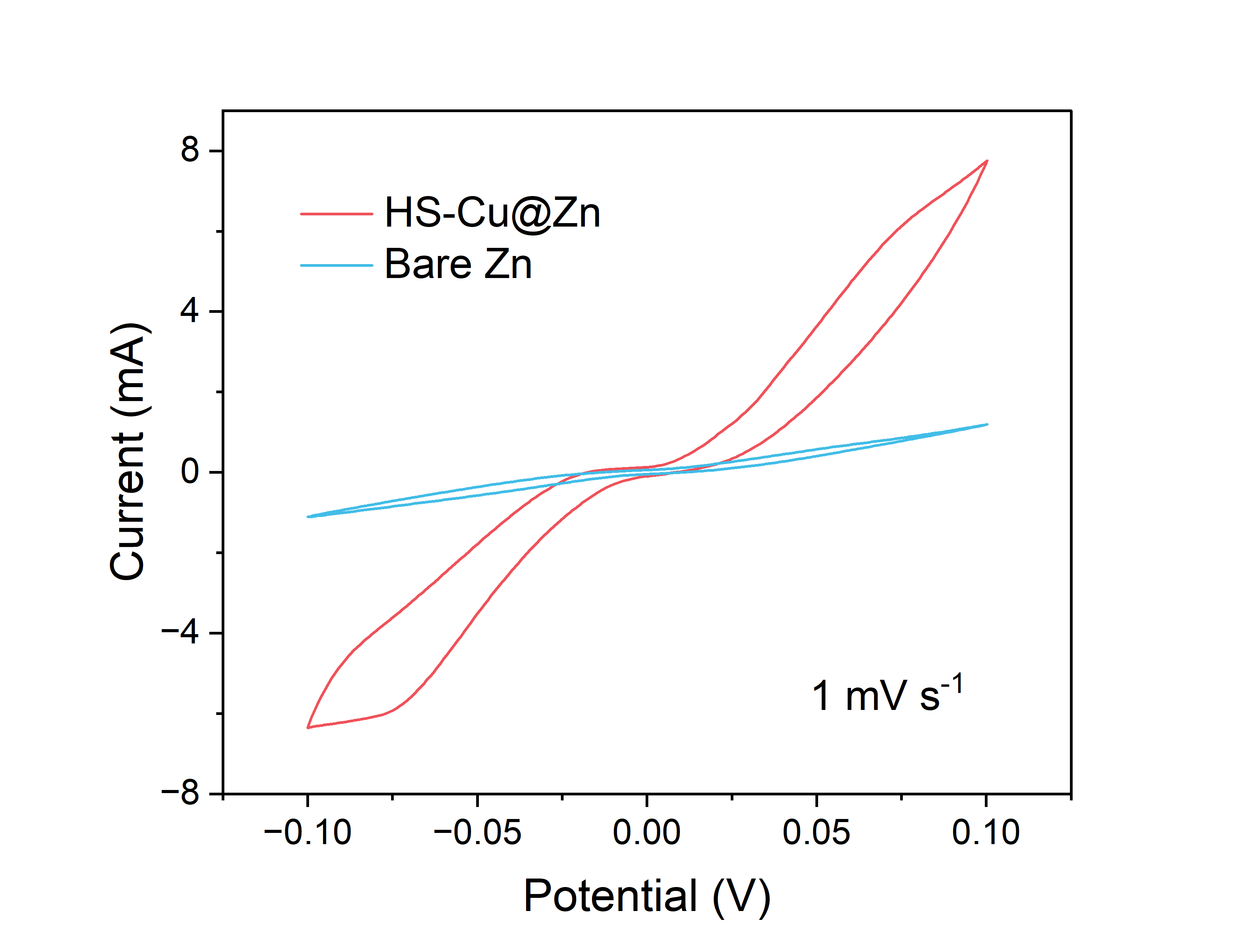


**Fig. S14** CV profiles of bare Zn and HS-Cu@Zn symmetric cells at 1 mV s^−1^.


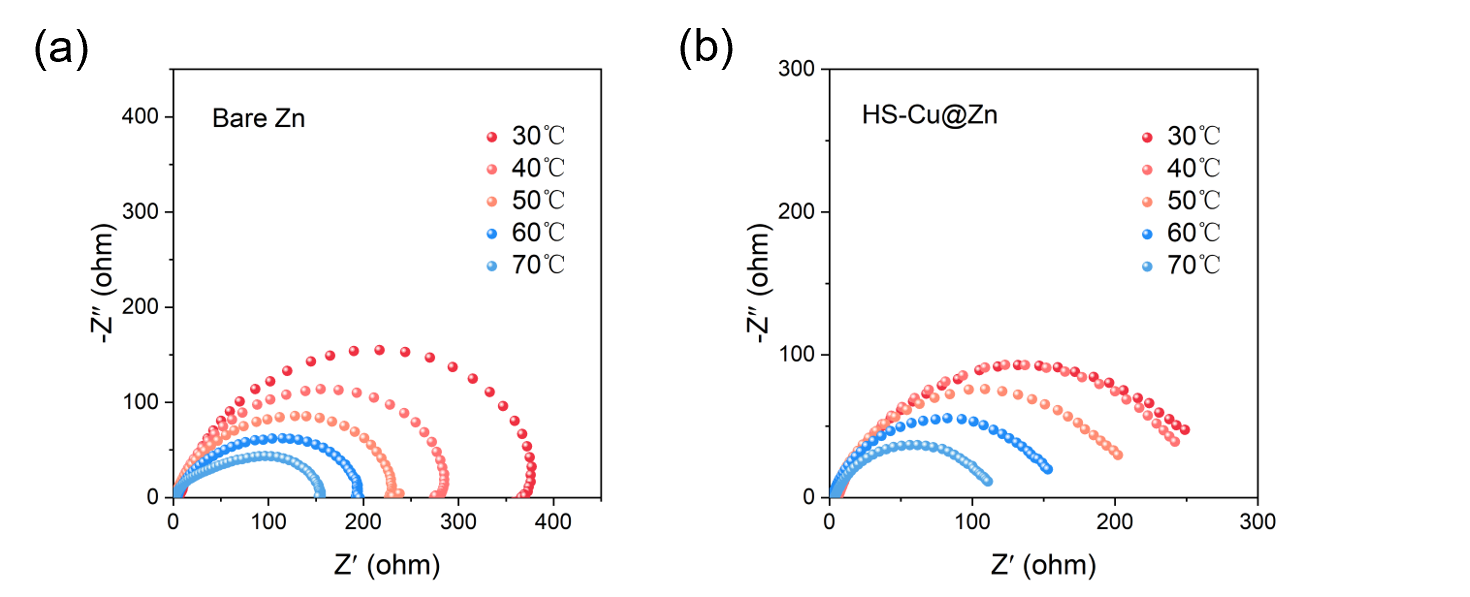


**Fig. S15** Nyquist plots at different temperatures for Zn||Zn symmetrical cells with (**a**) bare Zn anode and (**b**) HS-Cu@Zn anode


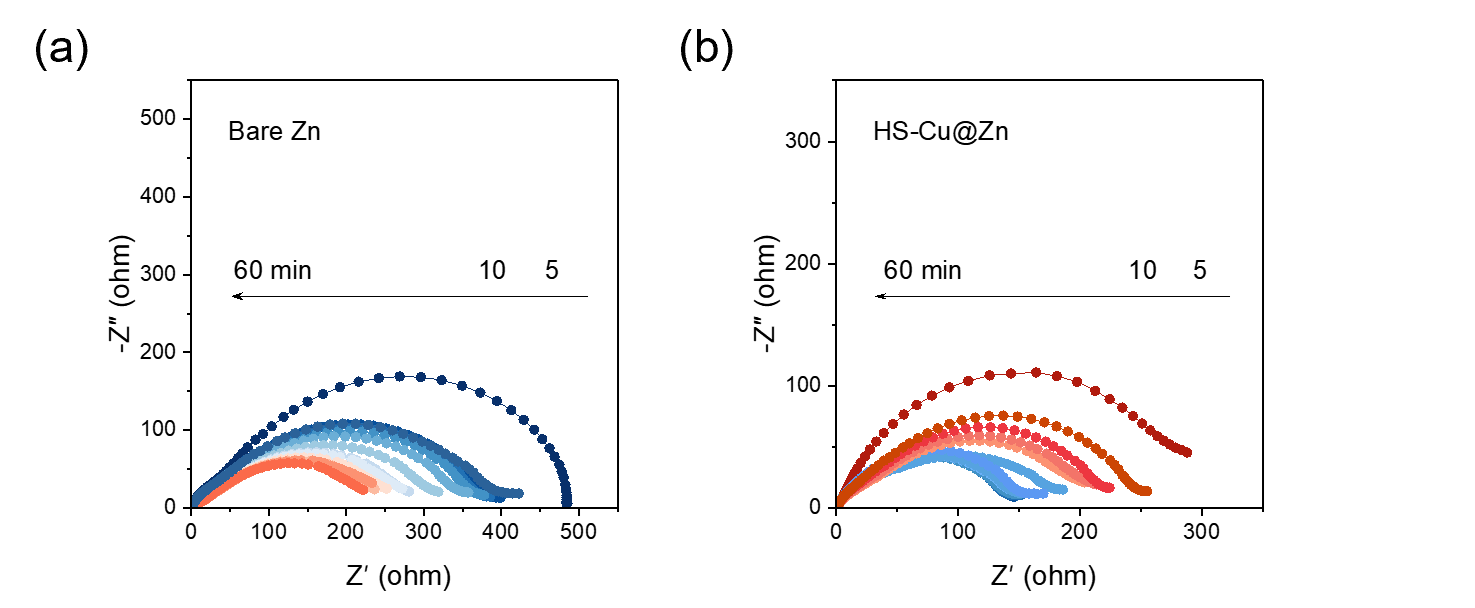


**Fig. S16** Operando EIS evolution of Zn||Zn symmetric cells using (**a**) bare Zn and (**b**) HS-Cu@Zn. The total Zn deposition time is 60 min


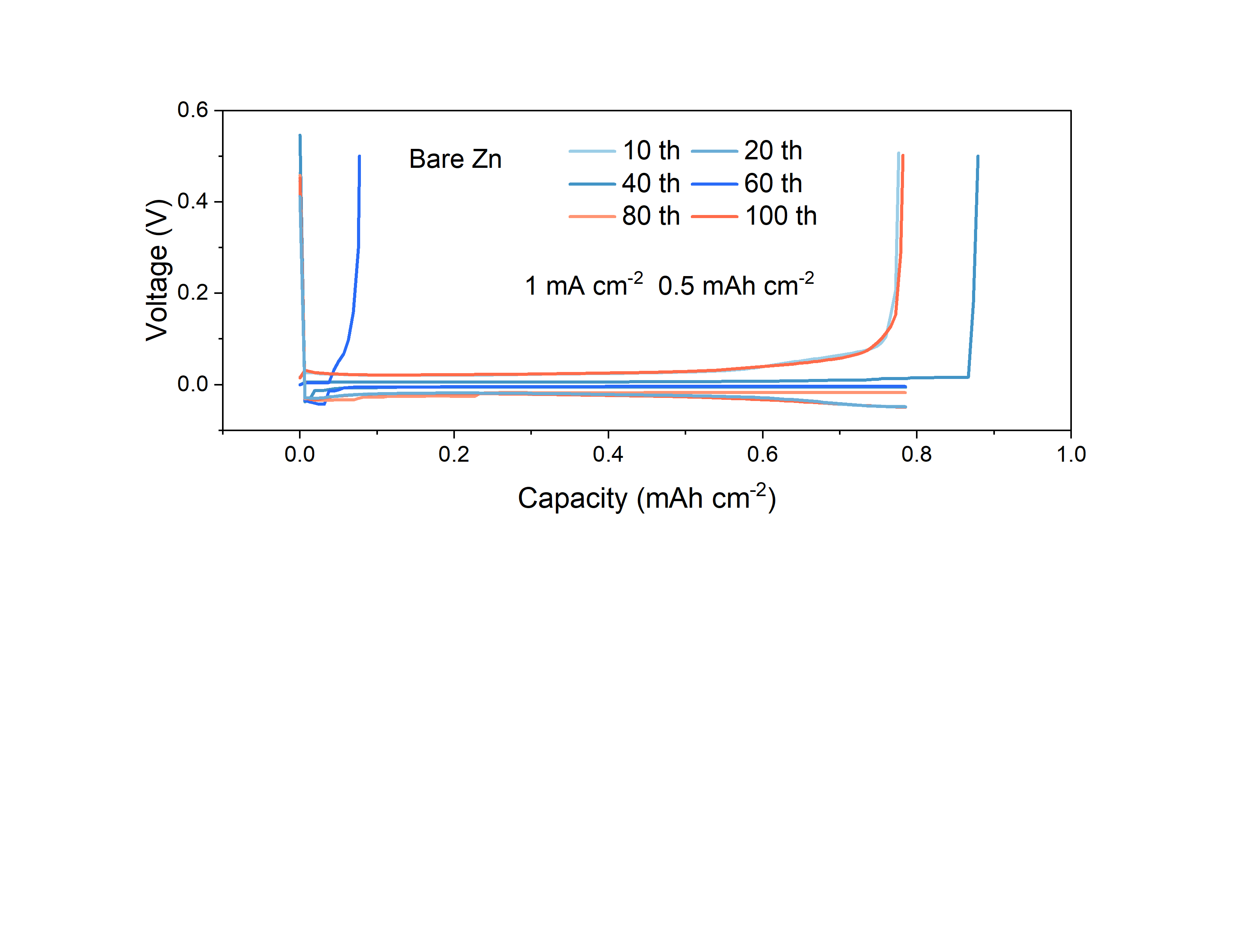


**Fig. S17** Polarization voltage curves of Cu||bare Zn cells at the 10th, 20th, 40th, 60th, 80th and 100th cycles, respectively


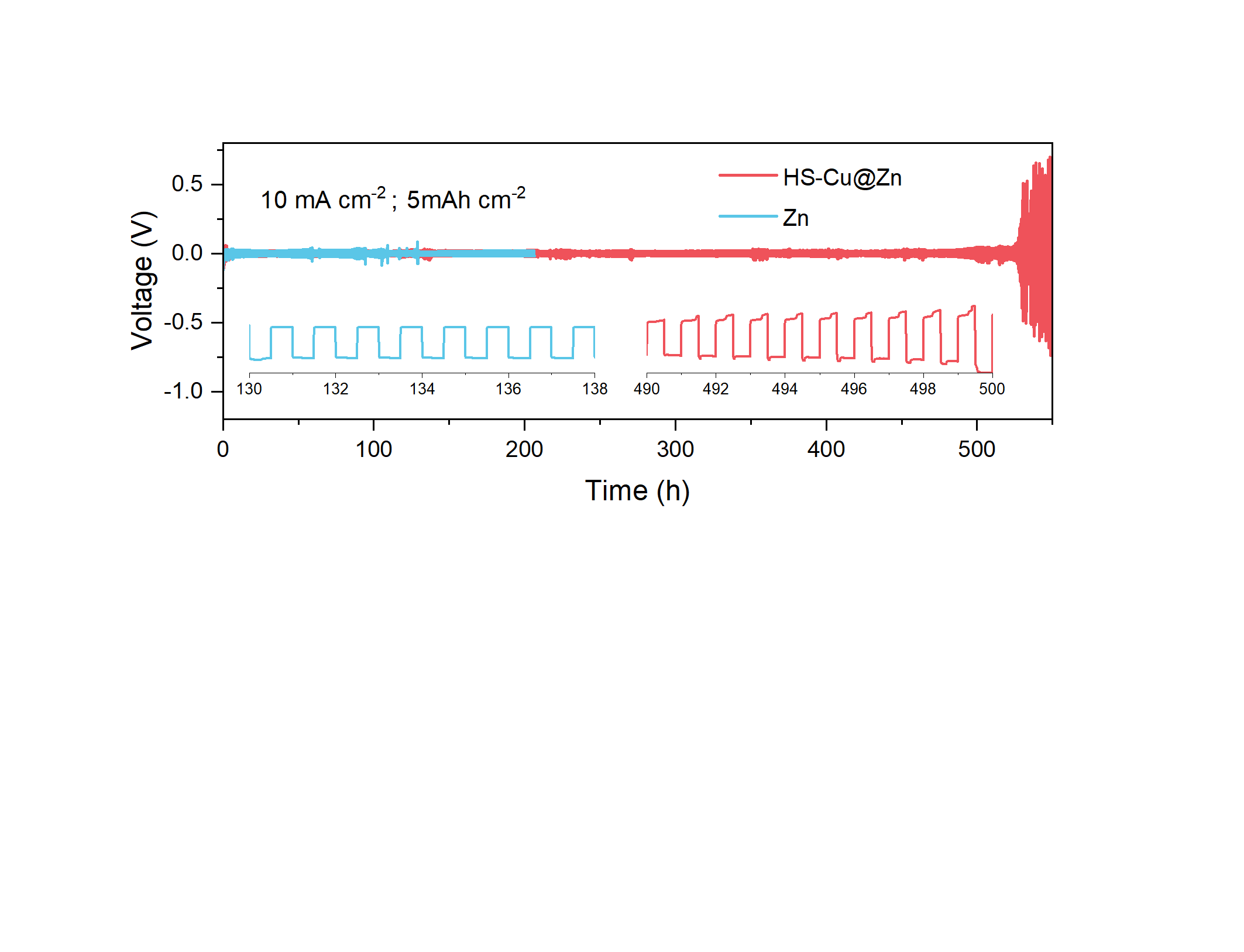


**Fig. S18** Cycling performance of bare Zn and HS-Cu@Zn symmetric cells at a high current density of 10 mA cm^−2^ for a capacity of 5 mAh cm^−2^


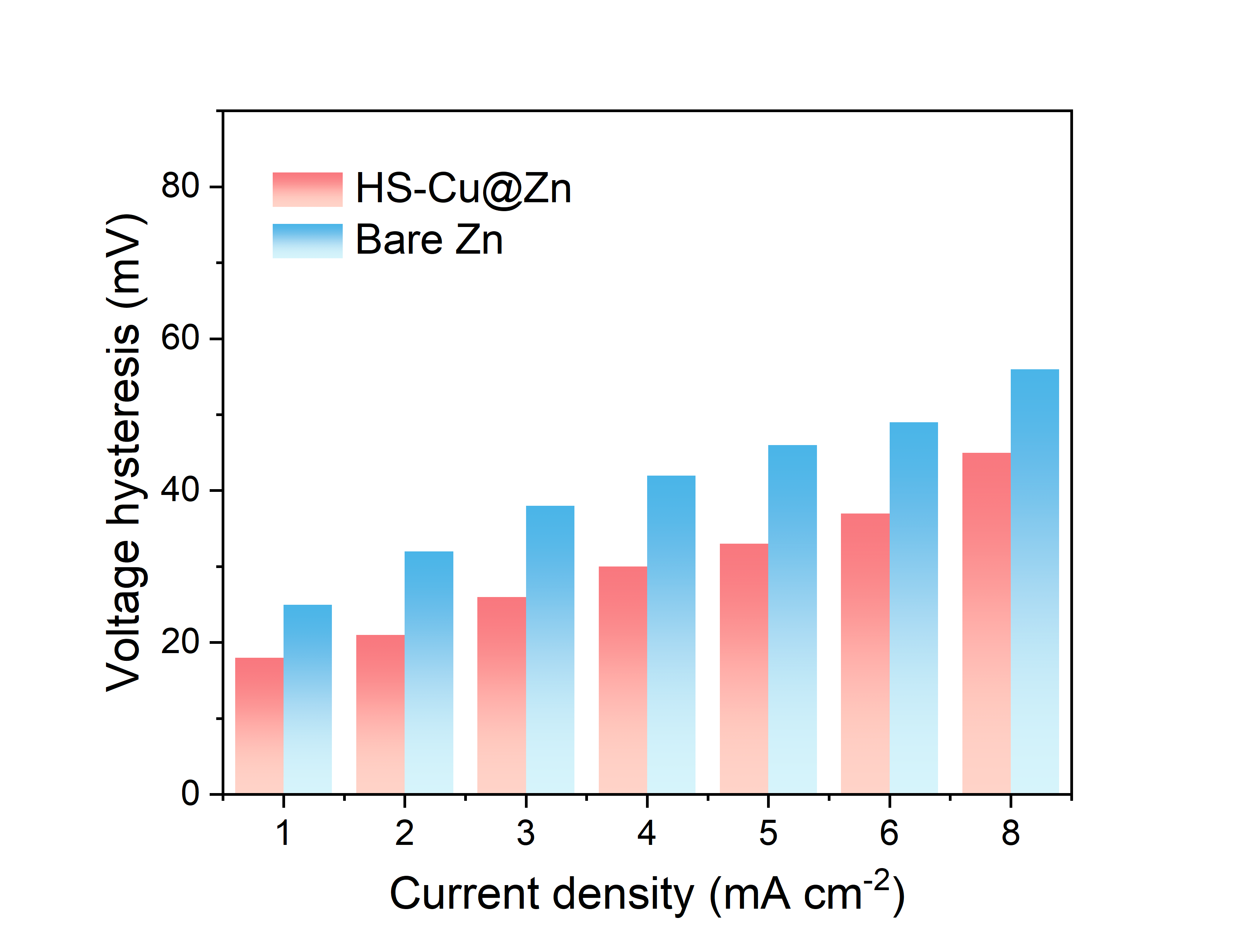


**Fig. S19** Voltage hysteresis of bare Zn and HS-Cu@Zn symmetric cells at different current densities ranging from 1 to 8 mA cm^–2^


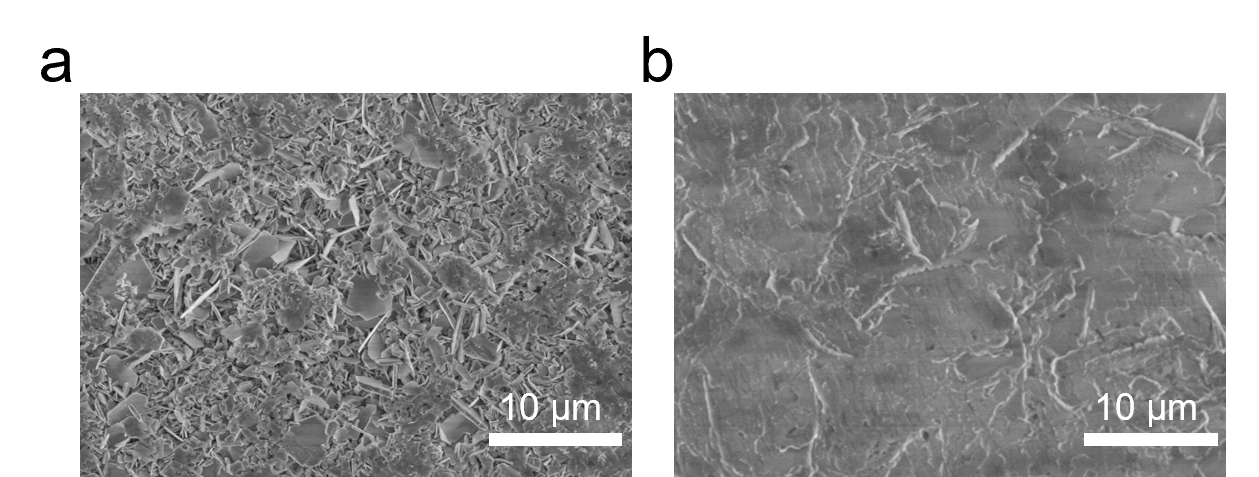


**Fig. S20** SEM images of the (**a**) bare Zn and (**b**) HS-Cu@Zn anodes after 20 cycles at 1 mA cm^−2^ for 1 mAh cm^−2^


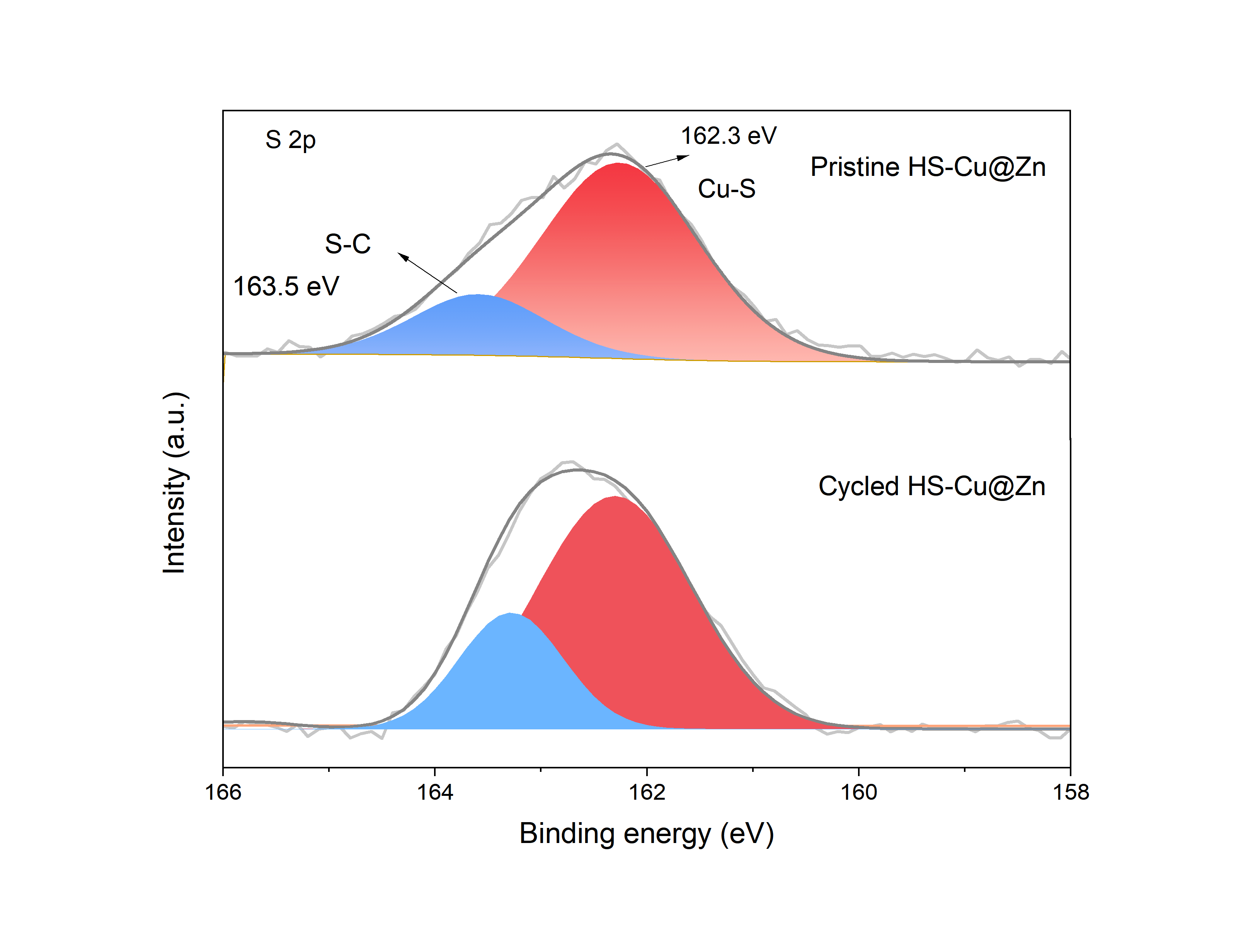


**Fig. S21** XPS profiles of pristine and cycled HS-Cu@Zn anodes in 2 M ZnSO_4_


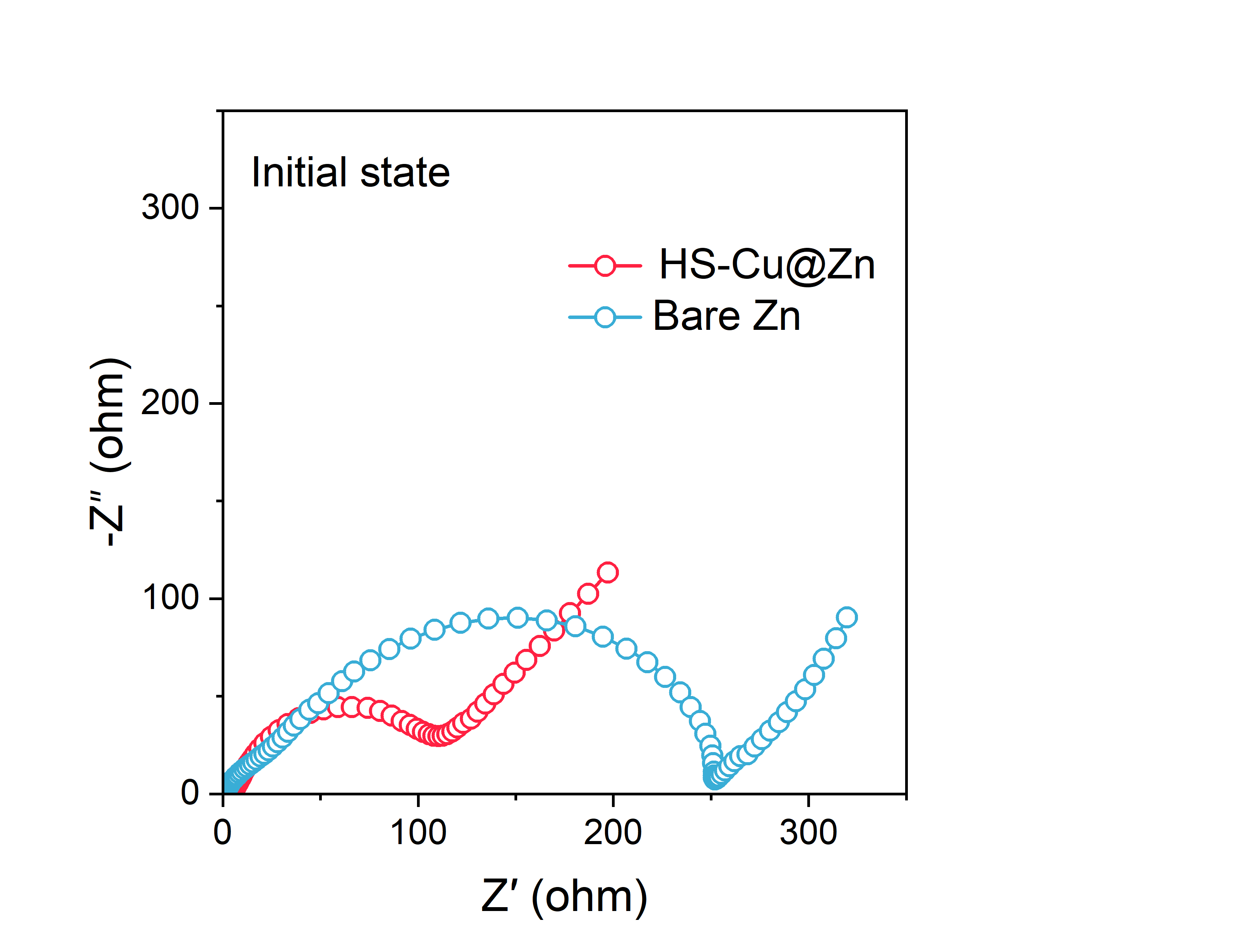


**Fig. S22** EIS results of ZnVO||bare Zn and ZnVO||HS-Cu@Zn full cells before cycling


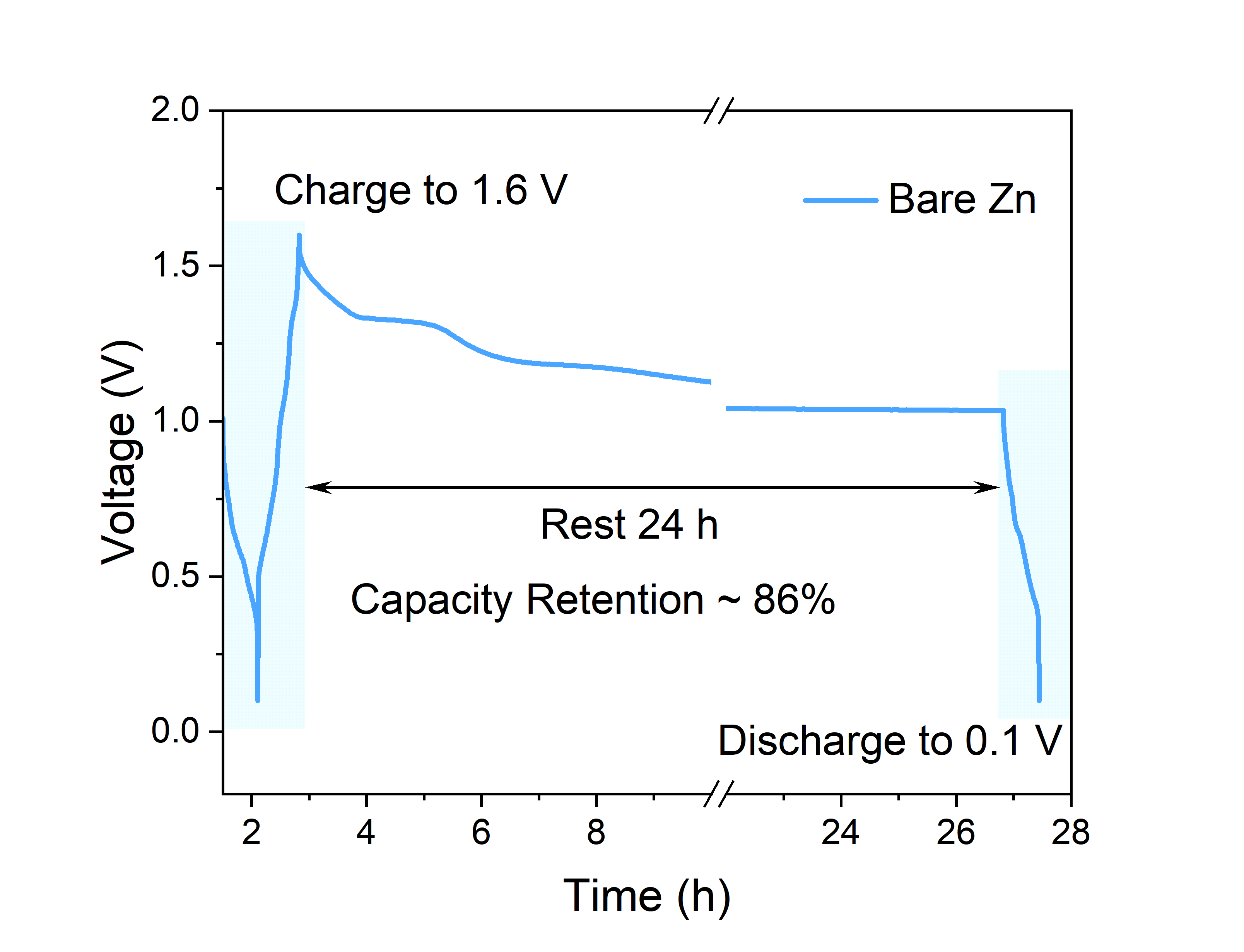


**Fig. S23** Capacity retention capabilities of ZnVO||bare Zn full cells after charging to 1.6 V, resting for 24 h, and discharging to 0.1 V

**Table S1** Comparison of HS-Cu@Zn anode with representative Zn interface designs

| **System** | **CE (%)** | **Symmetric Cell Stability (h)** | **Current Density (mA cm⁻^2^)** | **Zn^2+^ Transference Number** | **Desolvation E_a_ (kJ mol⁻^1^)** |
| --- | --- | --- | --- | --- | --- |
| **This work (HS-Cu@Zn)** | 99.65 (900 cycles) | >3500 | 1–10 | 0.75 | 18.04 |
| **[S1] q-P2VP-Zn**  **(Adv. Mater.)** | 97.8 (200 cycles) | 600 | 1-5 | 0.58 | \ |
| **[S2] BMC@Zn@In**  **(Adv. Funct. Mater.)** | 99.7 (500 cycles) | 2000 | 1-6 | \ | 21.6 |
| **[S3] 3D Zn-W**  **(Adv. Energy Mater.)** | 99.2 (300 cycles) | 2400 | 1-6 | 0.73 | 26.2 |
| **[S4] Zn-SHn**  **(Adv. Mater.)** | 99 (150 cycles) | 3500 | 1–10 | \ | \ |
| **[S5] TEOS/PDMS@Zn**  **(Adv. Funct. Mater.)** | 98.9 (60 cycles) | 1960 | 1–6 | 0.6 | 18.7 |
| **[S6] ZnF_2_-In@Zn**  **(ACS nano)** | 97.5 (30 cycles) | 4200 | 1–10 | 0.74 | 35.6 |

**Table S2** Coulombic Efficiency Comparison Between HS-Cu@Zn//ZnOV Batteries and Typical Zn Interface Designs

| **System** | **This work** | **[S7]HT-Zn** | **[S8]SA-coated Zn** | **[S9]CZ-Zn** | **[S10]BR-1@Zn** | **[S11]MA@Zn** |
| --- | --- | --- | --- | --- | --- | --- |
| CE (%) | 99.8% | 99.1% | 99.3% | 99.7% | 99.6% | 99.78% |

# Supplementary References

1. X. Cai, W. Tian, Z. Zhang, Y. Sun, L. Yang et al., Polymer coating with balanced coordination strength and ion conductivity for dendrite-free zinc anode. Adv. Mater. **36**(3), e2307727 (2024). <https://doi.org/10.1002/adma.202307727>
2. Z. Wang, J. Dong, K. Zhang, Z. Zhao, Y. Gao et al., Synergistic gradient design of a sandwich-structured heterogeneous anode for improved stability in aqueous zinc-ion batteries. Adv. Funct. Mater. **35**(37), 2505058 (2025). <https://doi.org/10.1002/adfm.202505058>
3. M. Lu, B.-H. Xiao, Y.-X. Lu, K. Xiao, Z.-Q. Liu, Structural design and interface modification with selective H+ binding of 3D zinc anode for aqueous zinc-ion batteries. Adv. Energy Mater. **15**(30), 2500785 (2025). <https://doi.org/10.1002/aenm.202500785>
4. J.-L. Yang, J. Li, J.-W. Zhao, K. Liu, P. Yang et al., Stable zinc anodes enabled by a zincophilic polyanionic hydrogel layer. Adv. Mater. **34**(27), e2202382 (2022). <https://doi.org/10.1002/adma.202202382>
5. W. Huang, D. Yan, Q. Li, Y. Lei, S. Yi et al., A chemical sewing enabled all-In-one control interface for robust zinc metal anodes. Adv. Funct. Mater. **34**(39), 2403196 (2024). <https://doi.org/10.1002/adfm.202403196>
6. X. Liu, Y. Zhang, L. Wang, R. Diao, T. Li et al., Highly reversible dendrite-free zinc anode enabled by a bilayered inorganic-metal interface layer. ACS Nano DOI:10.1021/acsnano.4c11486. (2024). <https://doi.org/10.1021/acsnano.4c11486>
7. B. Ren, S. Hu, A. Chen, X. Zhang, H. Wei et al., Inhibiting dendrite formation and electrode corrosion *via* a scalable self-assembled mercaptan layer for stable aqueous zinc batteries. Adv. Energy Mater. **14**(3), 2302970 (2024). <https://doi.org/10.1002/aenm.202302970>
8. H. Dong, X. Hu, R. Liu, M. Ouyang, H. He et al., Bio-inspired polyanionic electrolytes for highly stable zinc-ion batteries. Angew. Chem. Int. Ed. **62**(41), e202311268 (2023). <https://doi.org/10.1002/anie.202311268>
9. L. Zhou, F. Yang, S. Zeng, X. Gao, X. Liu et al., Zincophilic Cu sites induce dendrite-free Zn anodes for robust alkaline/neutral aqueous batteries. Adv. Funct. Mater. **32**(15), 2110829 (2022). <https://doi.org/10.1002/adfm.202110829>
10. F. Zhang, Q. Meng, J.-W. Qian, J. Chen, W.-X. Dong et al., Selective interface engineering with large π-conjugated molecules enables durable Zn anodes. Angew. Chem. Int. Ed. **64**(15), e202425487 (2025). <https://doi.org/10.1002/anie.202425487>
11. Z. Zhao, B. Guo, Y. Huang, X. Wang, J. Bao et al., Selective acid etching construction of high (101) texture zinc metal anodes for high-performance zinc ion batteries. Small **21**(18), 2501569 (2025). <https://doi.org/10.1002/smll.202501569>
